# Thermodynamic-driven supramolecular transition from nanofibers to nanospheres: morphology-dependent antibacterial specificity of herb medicines

**DOI:** 10.1186/s13020-025-01185-z

**Published:** 2025-09-25

**Authors:** Ji-chang Wei, Xiao-yu Lin, Yi-hang Zhao, Xin-ru Tan, Zhi-xia Wang, Yuan-yuan Li, Xue-mei Huang, Peng-long Wang

**Affiliations:** 1 School of Chinese Pharmacy, Beijing University of Chinese Medicine, Beijing, 102488 China; 2 Shanxi Traditional Chinese Medical Hospital, Taiyuan, 030024 China

**Keywords:** Thermodynamics, Morphology transition, Supramolecular, *Scutellariae Radix*, *Coptidis Rhizoma*, Antibacterial activity

## Abstract

**Background:**

*Scutellariae Radix* (SR) and *Coptidis Rhizoma* (CR) are classic drug pairs used in clinical practice for clearing heat and drying dampness, purging fire for removing toxin. By further studying the mechanism of compatibility of SR and CR from the perspective of thermodynamically driven supramolecular phase transition, we could reveal the interaction between its pharmacodynamic components, and provide scientific basis for improving TCM efficacy.

**Methods:**

The SR-CR and its main components baicalin-berberine (BA-BBR) were taken as the research objects. The morphology of the mechanically mixed samples was characterized by malvern particle size analyzer and scanning electron microscope. UHPLC-Q-Orbitrap HRMS technology was employed to analyze the material basis of each mechanically mixed sample. ITC was used to investigate the effect of temperature on the binding ability between SR and CR. The structural differences of supramolecules in different morphology were explored by molecular dynamics simulation. Finally, *in vitro* antibacterial models (*E. faecium* and *B. subtilis, S. aureus*) were used to evaluate the antibacterial activities of the mechanically mixed samples and non-targeted metabolomics was used to explore the differences in antibacterial mechanisms.

**Results:**

The mechanical mixtures formed nanofibers (NFs), while heating induced a transition to nanospheres (NPs). Molecular dynamics simulations revealed that enhanced hydrogen bonding and tighter molecular packing under thermal conditions drove this morphological shift. *In vitro* antibacterial assays and non-targeted metabolomics showed NPs exhibited superior inhibition against *Staphylococcus aureus* by disrupting amino acid biosynthesis and metabolism, whereas NFs suppressed *Bacillus subtilis* via physical entanglement and interfered with energy metabolism.

**Conclusion:**

Driven by thermal energy, the existence form of supramolecules changed from NFs to NPs and the morphology of the formed supramolecules was maintained during their interaction with bacteria, further affected the biological activity.

**Graphical Abstract:**

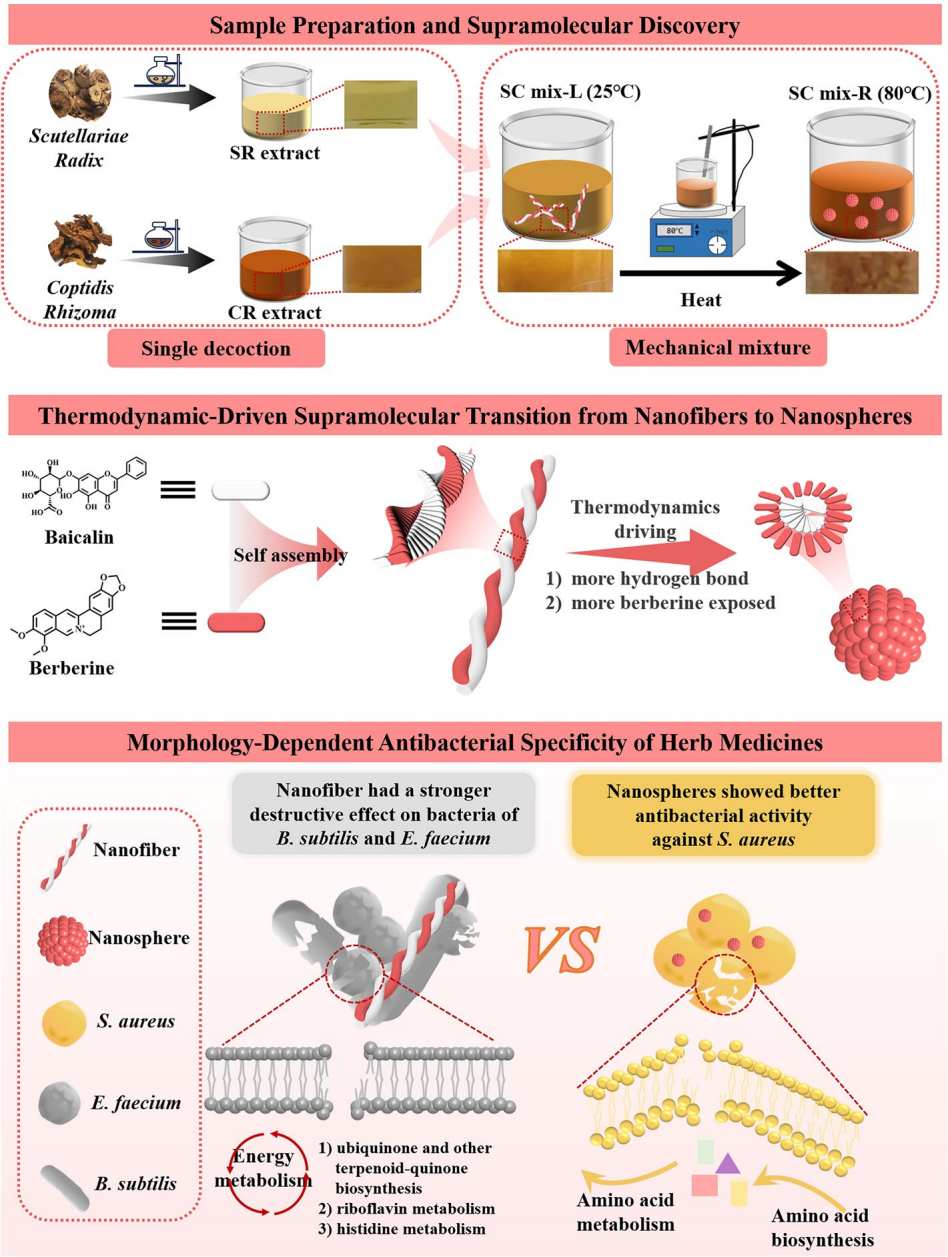

**Supplementary Information:**

The online version contains supplementary material available at 10.1186/s13020-025-01185-z.

## Introduction

The emergence of nanotechnology and the development of nanomedicine have greatly broadened the development of the medical field. However, the development of nanomedicine is greatly limited by the stability difference, complex preparation process and high cost [1, 2]. The emergence of supramolecular self-assembly technology has greatly promoted the research and development of nanomaterials [3]. The research of nano-Chinese herbal medicines (CHMs) has also become a hot spot in the industry because of its simple preparation, easy to obtain, safe, stable and reliable characteristics. Supramolecular system is a complex and ordered whole with certain integrity, microstructure and macro characteristics formed by the assembly of molecules through non-covalent bonds (hydrogen bonds, coordination bonds, electrostatic gravity or π–π stacking) [4]. Many natural star phytochemicals from CHMs have been successfully applied to various medical fields through self-assembly technology, including a variety of phytochemical components such as quinones, sugars, saponins, alkaloids, flavonoids, terpenoids and so on [5]. For example, celastrol and erianin were prepared into stable and uniform nanomedicine by self-assembly technology, which improved the therapeutic effect on breast cancer [6]. Supramolecular nanoparticles prepared by berberine (BBR) and chlorogenic acid self-assembly showed excellent inhibitory effect on *multi-drug resistant Staphylococcus aureus* [7]. BBR and hesperetin directly self-assemble to form binary carrier free multifunctional nanoparticles with synergistic anti-inflammatory activity, which has the potential to treat ulcerative colitis [5]. In addition, micelles and hydrogels obtained by self-assembly of phytochemical components have also shown great application prospects [8].

CHMs have garnered increasing attention for their synergistic therapeutic effects [9], yet the material basis and action mechanism underlying their bioactivity remains underexplored [10]. In recent years, more and more scholars have recognized that supramolecular system is an important effective form of CHMs decoction [11]. Supramolecular systems are ubiquitous in CHMs decoctions [12], such as QY305 [13], Danggui Buxue Decoction [14], Baihu Decoction [15], Huanglian Jiedu Decoction [16], Gegen Qinlian Decoction [17], Sanhuang Xiexin Decoction [18] and Maxing Shigan Decoction [17]. Supramolecular systems are widely used in the study of pharmacological activities and material basis of CHMs [11]. Therefore, the research method of supramolecular chemistry is helpful to establish the relationship between the macroscopic effect and the microscopic action form of CHMs.

*Scutellariae Radix* (SR) and *Coptidis Rhizoma* (CR) are classic drug pairs used in clinical practice for clearing heat and drying dampness, purging fire for removing toxin [19]. They are used in many prescriptions, such as Huanglian Jiedu Decoction and Gegen Qinlian Decoction. In the CHMs view, fungal and bacterial infection are external pathogen [20]. Exogenous pathogens entering the interior and turning into heat belong to the category of internal heat syndrome, and CHMs often treats it by heat-clearing and detoxicating medicinal, such as SR and CR [21]. Modern research showed that both SR and CR had antibacterial effects [22], especially the berberine from CR [23]. However, the material basis and effective form of their antibacterial action have not been fully revealed. Our previous study found that SR and CR showed obvious “precipitation” morphology when they were decocted together. Further studies found that the phenomenon was closely related to the formation of supramolecular system. For example, the main components baicalin (BA) and BBR of SR and CR could form spherical nanoparticles during the co-decoction process [24]. These nanoparticles exhibited significantly improved antibacterial activity and biofilm eradication against *Staphylococcus aureus* *(S. aureus)* compared to BBR [25]. Therefore, co-decoction is one of the necessary conditions for the formation of self-assembly process, which affects the clinical efficacy. What’s more, it should be noted that supramolecular self-assembly process is susceptible to external conditions, including solvent type [26, 27], pH [28, 29], temperature [30–32]. Based on the above research and questions, we propose the following research questions. What role does temperature play in the self-assembly process leading to the formation of supramolecular structures? What is the relationship between the formation and structural changes of supramolecular structures and the biological activity? These questions need further investigation.

The article focused on the relationship between the supramolecular differences and biological activity of the classical herb pair SR–CR and their main components BA and BBR driven by thermodynamics. The mechanical mixture of the drug pairs before and after heating showed different morphology states on the macroscopic scale. We found that the supramolecular system transforms from nanofibers (NFs) to nanospheres (NPs) under thermodynamic driving. Such transition from NFs to NPs further affected the antibacterial activity of the supramolecular system, as shown by the opposite antibacterial activity against *S. aureus, Bacillus subtilis (B. subtilis)* and *Enterococcus* *faecium* *(E. faecium)*. The results of the untargeted metabolomics analysis showed that thermodynamically driven morphology transition mediated selective antibacterial effects against different bacteria through modulation of core metabolic pathways.

## Materials and methods

### Materials

*Scutellaria baicalensis* Georgi (*Scutellariae Radix*, SR) and *Coptis chinensis* Franch. (*Coptidis Rhizoma*, CR) were purchased from Tong ren tang (Beijing, China). Berberine hydrochloride (BBR, C_20_H_18_ClNO_4_, 98%) and Baicalin (BA, C_21_H_18_O_11_, 98%) were purchased from Aladdin (China). Formic acid, phosphoric acid, acetonitrile and methanol were purchased from Fisher Company (USA). *Staphylococcus aureus* (*S. aureus*, ATCC 6538P) was acquired from school of Life Sciences, Beijing University of Chinese Medicine (Beijing, China). Live combined Bacillus subtilis and Enterococcus faecium granules with multivitamines was purchased from Beijing Hanmi Pharmaceutical Co., LTD (Beijing, China). Nutritional broth and nutritional agar were purchased from Solarbio biotechnology Co., Ltd (Beijing, China). PBS was purchased from Wuhan Servicebio Biotechnology Co., Ltd (Wuhan, China). Glutaraldehyde solution and ethanol were purchased from Beijing Chemical Plant (Beijing, China).

### Preparation of mechanical mixture of SR and CR

Firstly, SR or CR was weighed and immersed with 8 times the volume of water for 20 min, then refluxed for 15 min. After filtering out the residue, the SR single decoction (SS) and CR single decoction (CS) were obtained. Then, the single decoction was lyophilized (PILot5-8 M, Boyikang, China) for use.

The lyophilized powder of SS and CS was weighed at a ratio of 1:1. After dissolving with deionized water and mixing immediately, the SC mix-L was obtained.

The lyophilized powder of SS and CS was weighed at a ratio of 1:1. After dissolving with deionized water and heating in a water bath at 80 ℃ for 10 min, then mixing immediately and heating in 80 ℃ water bath for 30 min, the SC mix-R was obtained.

The above samples were prepared and freeze-dried for later use.

### Preparation of mechanical mixture of BA and BBR

BA and BBR were weighed and dispersed with water, respectively. Corresponding to SS and CS, respectively, BA-BBR mix-L and BA-BBR mix-R were prepared by the same preparation method as the mechanical mixture of SR and CR.

### Dynamic light scattering characterization (DLS)

Each sample was dispersed in deionized water at a same concentration and transferred to a cuvette. Malvern particle size analyzer (Zetasizer Nano ZEN3700, Maleven, UK) was used to measure the Zeta potential of each sample. The results were repeated three times and averaged.

### Scanning electron microscopy characterization (SEM)

The samples were dispersed in deionized water to the same concentration and 2 μL was transferred to silicon wafers for drying naturally at room temperature. After spraying gold on the surface (working voltage was 10.0 kV), the morphology and particle size of each sample was observed by SEM (Hitachi SU-8020, Hitachi, Japan).

### UV − Visible absorption spectrometric determination (UV–Vis)

Each sample with the same concentration was prepared. The detection wavelength of the UV-visible spectrophotometer was set at 200−600 nm and deionized water was used as a blank solution for full-wavelength scanning (UH5300, Hitachi, Japan).

### Fourier transform infrared spectroscopy determination (FT-IR)

The samples were put into a Fourier transform infrared spectrometer. Later, the infrared spectra of each sample were measured in the range of 400–4000 cm^−1^ with air as the background (ensor27, Bruker, USA).

### UHPLC-Q-orbitrap HRMS analysis

UHPLC-Q-Orbitrap HRMS analysis was performed using a UltiMate 3000 liquid chromatographic system and coupled with a Q Exactive quadrupole-Orbitrap high-resolution mass spectrometry (Thermo Fisher Scientifc, Massachusetts, USA). Each sample was prepared at 1 mg·mL^−1^ (based on the concentration of the lyophilized powder of single decoction) and filtered through 0.22 μm microporous filter membrane. TC-C18 column (4.6 mm × 250 mm, 5 μm) was used. The flow rate of mobile phase was maintained at 0.3 mL/min with 0.1% (v/v) aqueous formic acid solution (A) and acetonitrile (B). The gradient elution conditions were set as follows: 0–20 min, 2–98% B. The injection volume was 5 μL. The ion source adopted ESI to collect information in positive (ESI^+^) and negative (ESI^−^) electrospray ionization mode. Spray voltage was ± 3.5 kV. Lens voltage was 50 V. The pressure of sheath gas (N_2_) and auxiliary gas (N_2_) were adjusted to 40 arb and 0 arb, respectively. The temperature of ion transfer tube was 320 ℃. The temperature of the auxiliary device was 350 ℃. The scanning mode was Full MS, the resolution was 10,000, and the scanning range was 150–1500.

### Isothermal titration calorimetry determination (ITC)

SS and CS was prepared as reserve fluid, and SS was diluted five times with deionized water. All reserve fluid must be filtered with a 0.22 μm microporous membrane before use. The titration condition was CS titrated SS. The instrument parameters were set as follows: the stirring rate was 250 r/min; titration temperature was 25 °C, 37 ℃ and 50 ℃; the titration interval was 180 s (Nano ITC, TA, USA).

### Molecular dynamics simulation (MD)

Molecular dynamics (MD) simulations were performed using the Gromacs 2020.6 [33] package under periodic boundary conditions. The GAFF force-field [34] was employed, and the TIP3P model was used to describe the water molecule. The computational content mainly included energy minimization by conjugate gradient method, annealing equilibrium (0–100 ps, 0–298.15 K) in NPT ensemble for a total of 200 ps, and kinetic simulation at 298.15 K and 353.15 K for 50 ns. A cubic box with a side length of 6 nm was constructed, and 40 baicalin and 40 berberine molecules were added to the model. The acquisition of organic molecular topological files and the identification of atomic charges relied on Sobtop and Multiwfn [35, 36]. The figures were produced by the VMD software [37]. The gmx energy program was used to calculate the short-range Lennard–Jones interaction energies E_(LJ-SR)_ and Coulomb interaction energies E_(Coul-SR)_, the gmx analyze program was used to calculate the average number of hydrogen bonds. The gmx sasa and gmx rms program was used to indicate that the simulations were converged.

### *In vitro* antibacterial experiment

The antibacterial experiments against *B. subtilis*, *E. faecium* and *S. aureus* were carried out by dilution coating plate method. The antibacterial activities of SC mix-L and SC mix-R were compared, and CS was used as a positive control. Samples were diluted into drug suspensions in a 48-well plate with a final volume of 500 μL per well, and finally 50 μL of bacterial suspension was added to each well separately. The plate was placed in a constant thermostatic incubator at 37 ℃ for 16 h. After that, the bacterial suspension was diluted with nutrient broth 1 × 10^5^ times, then inoculated on nutrient AGAR medium, and cultured in constant thermostatic incubator at 37 °C for 16 h. The final concentrations of drug suspension were 8, 6, and 4 mg·mL^−1^, and the concentrations of bacterial suspension were 1.35 × 10^4^ CFU·mL^−1^ for *E. faecium* and 1.5 × 10^3^ CFU·mL^−1^ for *B. subtilis.* The experiment was repeated three times.

The antibacterial activity against *S. aureus* was compared using the same experimental method. And the final concentration of drug suspension was 0.1, 0.08 and 0.05 mg·mL^−1^, and the concentrations of bacterial suspension were 2 × 10^6^ CFU·mL^−1^*.*

### Live and dead bacteria staining

After 1 mL nutrient broth medium was added to a 12-well plate, 100 μL Bacillus subtilis double live suspension (*E. faecium* 1.35 × 10^4^ CFU·mL^−1^ and *B. subtilis* 1.5 × 10^3^ CFU·mL^−1^) were added. The plate was placed in a constant thermostatic incubator at 37 ℃ for 12 h. According to the above experimental method of bacterial culture, the cells were diluted with nutrient broth to final concentrations of 8 mg·mL^−1^ and continued to be incubated for 12 h at 37 ℃ in a constant temperature biochemical incubator. After the nutrient broth was discarded, Live/Dead BacLight^™^ reagent was added to the wells. The laser confocal microscope (Leica TCSSP8, Leica, Germany) was used to take photos without light.

The same experimental method was used to compare the damage to the *S. aureus*. The cells were diluted to a final concentration of 0.1 mg·mL^−1^ with nutrient broth medium, and the other experimental procedures were the same.

### Morphological characterization of bacteriostatic experiments in vitro

SEM was used to observe the morphological changes of the bacteria treated with samples. The bacteria were incubated in the same way as live and dead bacteria staining. Then the bacterial liquid of each sample was collected in different centrifuge tubes, centrifuged (3000 r/min, 10 min) to collect the bacteria. Each centrifuge tube was fixed at 4 °C for 4 h after adding 2.5% glutaraldehyde. And then eluted with ethanol solution with gradient concentration of 30, 50, 70, 80, 90 and 100%, respectively (3000 r/min, 10 min each time). After gold spraying, bacteria were observed under SEM.

### Non-targeted metabonomics analysis

Sample Preparation and Detection: Bacterial were collected following the methods for SEM sample preparation. The bacteria were resuspended in 1 mL of pre-cooled PBS (4 °C) and centrifuged twice (3000 r, 5 min, 4 °C) to remove residual nutrient broth and drug suspensions. Subsequently, 1 mL of PBS and 3 mL of methanol were added, followed by vortex mixing and ultrasonication at 15 °C for 1 h. The supernatant was collected after centrifugation (3000 r, 5 min, 4 °C), concentrated to dryness under nitrogen stream, and redissolved with a methanol-ultrapure water mixture (1:3, v/v). The solution was filtered through a 0.22 μm microporous filter membrane for analysis. Quality control (QC) samples were prepared by mixing equal volume from each sample. Each group has six samples repeated. For more information, see the supplementary material.

Data processing and statistical analysis: Raw data were preprocessed using Progenesis QI software (Waters MS Technologies, Manchester, UK) for peak alignment, retention time correction, and feature extraction. MetaboAnalyst 6.0 was employed for data filtering, missing value imputation, fold change (FC) calculation, and normalization. Multivariate statistical analyses, including principal component analysis (PCA) and partial least squares-discriminant analysis (PLS-DA), were performed using MetaboAnalyst 6.0. Potential differential metabolites were screened based on thresholds of P-value (< 0.05), variable importance in projection (VIP > 1), and fold change (FC > 2 or FC < 0.5). Pathway enrichment analysis of the identified metabolites was conducted via MetaboAnalyst 6.0 using the KEGG database.

### Statistical analysis

Quantitative data were expressed as the means ± standard deviation of at least three independent assays. Significance analysis was evaluated by one-way analysis of variance, ANOVA. *P* < 0.05 was considered statistically significant.

## Results and discussion

### Supramolecular formation and morphological characterization

**Fig. 1:**
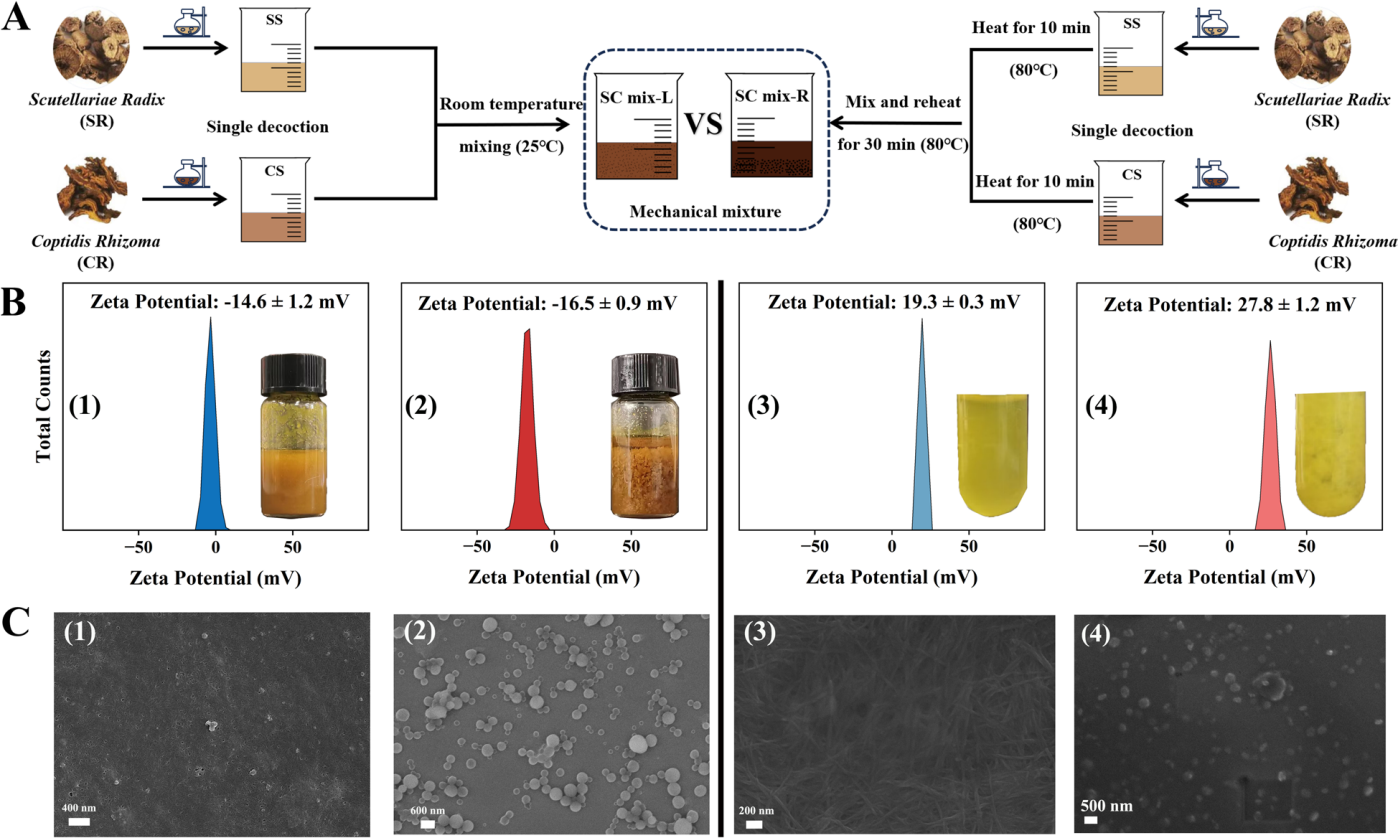
Morphological characterization of SC mix-L (1), SC mix-R (2), BA-BBR mix-L (3) and BA-BBR mix-R (4). **A** Preparation of SC mix-L and SC mix-R. **B** Dynamic light scattering characterization. **C** Scanning electron microscopy characterization

During the process of sample preparation (Fig. 1A), we found that the mixing methods had a great influence on the macro/micro morphology of the sample. Specifically, direct mixing of SS and CS could produce obvious turbidity, but different mixing methods had differences in apparent morphology. The precipitation and agglomeration of the heated system after mixing (SC mix-R) were more obvious than SC mix-L. Correspondingly, BA and BBR, the main components of the drug pair, also showed a similar morphology transition, showing that the precipitation and agglomeration formed by the heated system after mixing (BA-BBR mix-R) was more obvious.

To further investigate the effect of the thermal energy on the morphology transition, the microscopic morphology of each sample was characterized by SEM. As shown in Fig. 1B, C(1) and (2), the morphology of SC mix-L was NFs. The morphology of the heated sample SC mix-R was NPs, and the dispersion was relatively uniform. In addition, when mixed with heated SS and CS, the microscopic morphology showed a transition state between NFs and NPs (Fig. S1). The results of SEM showed the formation of supramolecular system and the occurrence of morphology transition at the microscopic level. Combined with the DLS characterization results, it was found that this morphology transition was reflected in the enhanced morphology stability (the absolute value of Zeta potential) of the sample system after heating. SC mix-L had the smaller absolute value of Zeta potential (14.6 mV) and SC mix-R had the larger absolute value of Zeta potential (16.5 mV). Similarly, the supramolecular system formed by the main pharmacodynamic ingredients BA and BBR was observed, and the observed changes exhibited the same trend. As shown in Fig. 1B, C(3) and (4), the morphology of BA-BBR mix-L was NFs and the morphology of the heated sample BA-BBR mix-R was NPs. Correspondingly, the absolute value of Zeta potential of the sample system increased after heating. The absolute value of Zeta potential of BA-BBR mix-L was smaller (19.3 mV), and the absolute value of Zeta potential of BA-BBR mix-R was larger (27.8 mV).

Based on the above results, we found that thermal energy could promote the morphology transition of SR-CR at the macro/micro level and affect the existence state of the supramolecular system. However, the mechanism of this transition and its effect on biological activity remain to be further explored.

### The mechanism of supramolecular formation

The spectral properties of the supramolecule were investigated by UV–vis and FT-IR. The results of UV–vis absorption spectra revealed that the characteristic absorption peaks of the two SC mix samples were the same with no significant difference observed. There were two absorption peaks around 275 nm and 330 nm, suggesting that the conjugated system did not change significantly upon heating. The results of infrared spectrum analysis further indicated that there was no significant difference and change in peak types, suggesting that the action sites of SR-CR did not change significantly with heating. Similarly, the spectroscopic characterization results of the BA-BBR mix samples were found to be consistent with those obtained at the SC mix samples.

**Fig. 2:**
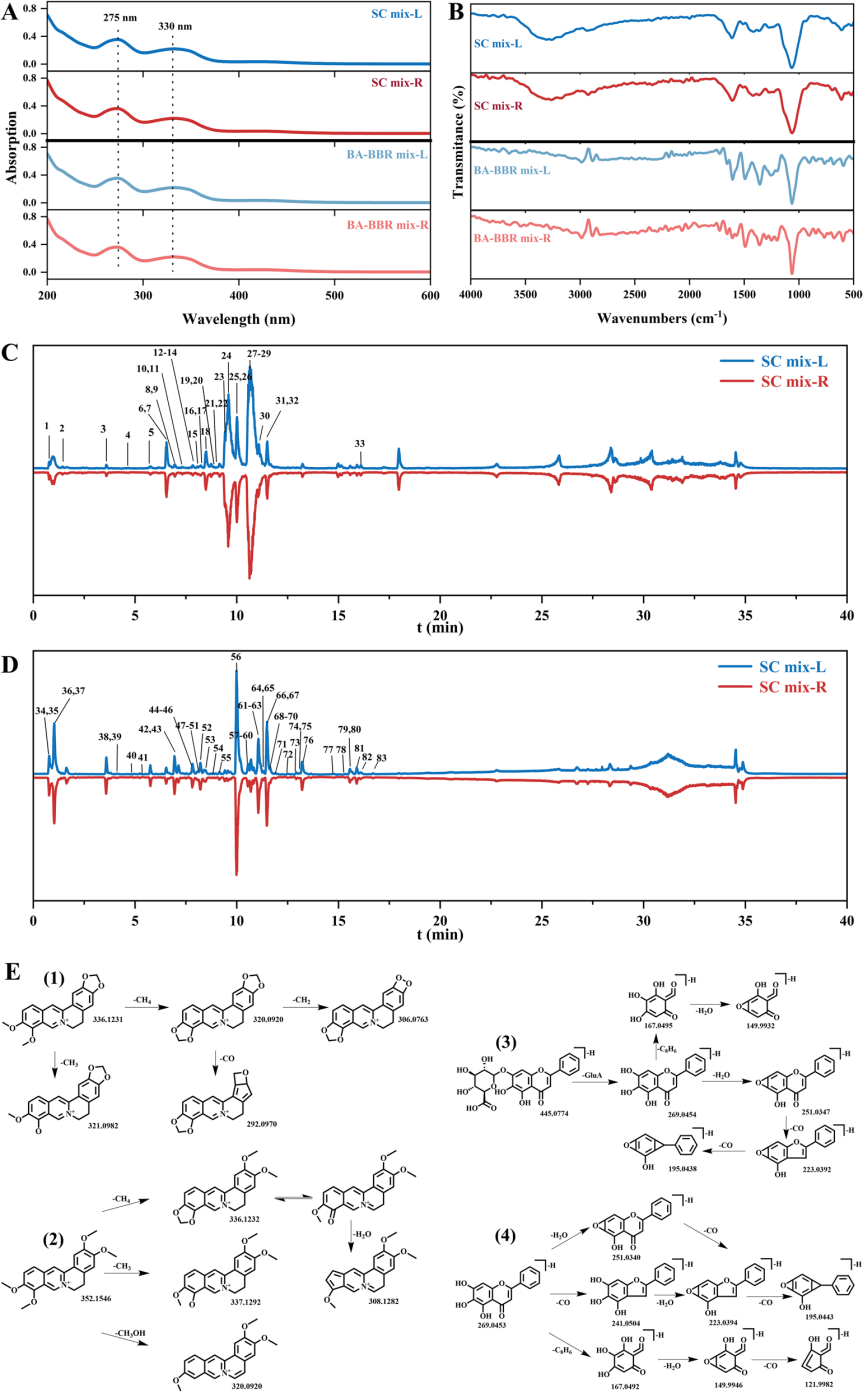
**A** UV − visible spectra. **B** Fourier infrared spectra. **C** Total ion current chromatograms in the positive ion modes. **D** Total ion current chromatograms in the negative ion modes. **E** Fragmentation pathway of (1) berberine (2) palmatine (3) baicalin and (4) baicalein

In order to explore the chemical composition differences of different samples, UHPLC-Q-Orbitrap HRMS was used to analyze the chemical composition of each sample in positive and negative ion modes. A total of 83 compounds were identified from the two samples (Fig. 2C, D), including 26 alkaloids, 41 flavonoids and 16 other compounds (Table 1). Alkaloids were the main medicinal ingredients of CR, the fragmentation pathway was shown in Fig. 2E(1) and (2). Compounds 27 and 29 were characterized to be palmatine and berberine, respectively. Compound 29 (berberine) exhibited a molecular ion at *m/z* 336.1231 and its fragment ions were [M-CH_3_]^+^ (*m/z* 321.0982), [M-CH_4_]^+^ (*m/z* 320.0920), [M-CH_4_-CO]^+^ (*m/z* 292.0970) and [M-CH_4_-CH_2_]^+^ (*m/z* 306.0763). Compound 27 (palmatine) exhibited a molecular ion at *m/z* 352.1546 and its fragment ions were [M-CH_3_]^+^ (*m/z* 337.1292), [M-CH_4_]^+^ (*m/z* 336.1232), [M-CH_4_-H_2_O]^+^ (*m/z* 308.1282) and [M-CH_3_OH]^+^ (*m/z* 320.0920). Flavonoids were the main medicinal ingredients of SR, the fragmentation pathway of representative components baicalin and baicalein were shown in the Fig. 2E(3) and (4), respectively. Compound 56 was characterized to be baicalin and it exhibited a molecular ion at *m/z* 445.0774. Its fragment ions were [M-H-GluA]^−^ (*m/z* 269.0454), [M-H-GluA-H_2_O]^−^ (*m/z* 251.0347), [M-H-GluA-H_2_O-CO]^−^ (*m/z* 223.0392), [M-H-GluA-H_2_O-CO-CO]^−^ (*m/z* 195.0438), [M-H-GluA-C_8_H_6_]^−^ (*m/z* 167.0495) and [M-H-GluA-C_8_H_6_-H_2_O]^−^ (*m/z* 149.9932). Compound 76 was characterized to be baicalein and it exhibited a molecular ion at *m/z* 269.0453. Its fragment ions were [M-H-H_2_O]^−^ (*m/z* 251.0340), [M-H-CO]^−^ (*m/z* 241.0504), [M-H-H_2_O-CO]^−^ (*m/z* 223.0394), [M-H-H_2_O-CO-CO]^−^ (*m/z* 195.0443), [M-H-C_8_H_6_]^−^ (*m/z* 167.0492), [M-H-C_8_H_6_-H_2_O]^−^ (*m/z* 149.9946) and [M-H-C_8_H_6_-H_2_O-CO]^−^ (*m/z* 121.9982). The results of UHPLC-Q-Orbitrap HRMS analysis indicated that the chemical composition of the mechanical mixture samples was the same, which was composed of components from SR and CR. Additionally, HPLC results demonstrated the content of major active compounds BA and BBR in SC mix-L and SC mix-R were the same (Fig. S2). This suggested that different mixing methods had no effect on the composition of the sample components.

**Table 1:** Identification of Compounds

No	t_R_ (min)	Compand	Formula	Identity	Theoretical (m/z)	Experimental (m/z)	Δppm	Fragment ion (m/z)	Source
1	0.89	Arginine	C_6_H_14_N_4_O_2_	[M + H]^+^	175.1190	175.1191	0.57	158.0925, 157.1086, 140.0824, 116.0709, 112.0873	SR,CR
2	1.47	Phenylalanine	C_9_H_11_NO_2_	[M + H]^+^	166.0863	166.0865	1.20	120.0240	SR,CR
3	3.59	Caffeic acid	C_9_H_8_O_4_	[M + H]^+^	181.0495	181.0496	0.55	163.0390	SR,CR
4	4.66	Tryptophan	C_11_H_12_N_2_O_2_	[M + H]^+^	205.0972	205.0974	0.98	161.1078	SR,CR
5	5.66	Magnocurarine	C_19_H_24_NO_3_^+^	[M]^+^	314.1756	314.1752	− 1.27	269.1172, 237.0900	CR
6	6.42	9-O-Berberine glucoside	C_25_H_26_NO_9_^+^	[M]^+^	484.1608	484.1606	− 0.41	322.1091, 308.0918	CR
7	6.55	Magnoflorine	C_20_H_24_NO_4_^+^	[M]^+^	342.1705	342.1705	0.00	297.1124, 282.0891, 265.0861, 237.0910	CR
8	6.85	11-Hydroxyl-groenlandicine	C_19_H_16_NO_5_^+^	[M]^+^	338.1028	338.1024	− 1.18	322.0710, 310.1073, 306.0752	CR
9	6.96	11-Hydroxy-stepholidine-glucoside	C_25_H_29_NO_10_	[M + H]^+^	504.1864	504.1864	0.00	342.1345, 188.0619	CR
10	7.19	Magnoflorine glucoside	C_26_H_34_NO_9_^+^	[M]^+^	504.2234	504.2237	0.59	342.1700	CR
11	7.29	8,9-Di-demethyl-epiberberine	C_18_H_14_NO_4_^+^	[M]^+^	308.0923	308.0919	− 1.30	280.0970, 278.0808	CR
12	7.70	8-Oxocoptisine	C_19_H_13_NO_5_	[M + H]^+^	336.0866	336.0869	0.89	318.0735, 308.0919, 294.0770	CR
13	7.71	Dihydrojatrorrhizine	C_20_H_22_NO_4_^+^	[M]^+^	340.1549	340.1545	− 1.18	325.1292, 324.1232	CR
14	7.90	Menisperine	C_21_H_26_NO_4_	[M]^+^	356.1862	356.1856	− 1.68	311.1277	CR
15	8.05	Demethyleneberberine glucoside	C_25_H_28_NO_9_	[M]^+^	486.1764	486.1766	0.41	324.1234, 308.0912	CR
16	8.17	Noroxyhydrastinine	C_10_H_9_NO_3_	[M + H]^+^	192.0655	192.0657	1.04	149.0600	CR
17	8.21	Viscidulin I	C_15_H_10_O_7_	[M + H]^+^	303.0499	303.0502	0.99	153.0186	SR
18	8.49	Demethylenepiberberine	C_19_H_18_NO_4_^+^	[M]^+^	324.1236	324.1226	− 3.09	309.1005, 294.0756, 266.0809	CR
19	8.85	Dihydropalmatine	C_21_H_23_NO_4_	[M + H]^+^	354.1700	354.1701	0.28	338.1379, 324.1235	CR
20	8.94	Quercetin	C_15_H_10_O_7_	[M + H]^+^	303.0499	303.0502	0.99	257.0452, 229.0496, 153.0118	SR
21	9.03	Oxyepiberberine	C_20_H_17_NO_5_	[M + H]^+^	352.1179	352.1190	3.12	336.0881, 322.0716, 308.0930	CR
22	9.07	Columbamine	C_20_H_20_NO_4_^+^	[M]^+^	338.1392	338.1392	0.00	322.1078, 294.1126, 308.0921, 294.1126	CR
23	9.42	Jatrorrhizine	C_20_H_20_NO_4_^+^	[M]^+^	338.1392	338.1386	− 1.77	322.1074, 308.0901, 306.1136, 294.1143	CR
24	9.63	Coptisine	C_19_H_14_NO_4_^+^	[M]^+^	320.0923	320.0919	− 1.25	292.0968	CR
25	10.11	Berberrubine	C_19_H_16_NO_4_^+^	[M]^+^	322.1079	322.1074	− 1.55	307.0818, 294.1133	CR
26	10.21	Viscidulin III	C_17_H_14_O_8_	[M + H]^+^	347.0761	347.0762	0.29	332.0528, 317.0296, 314.0422, 289.0346, 286.0469, 169.0133, 150.0314, 137.0243	SR
27	10.57	Palmatine	C_21_H_22_NO_4_^+^	[M]^+^	352.1548	352.1546	− 0.57	336.1232, 337.1292 322.1076, 308.1282	CR
28	10.70	Epiberberine	C_20_H_18_NO_4_^+^	[M]^+^	336.1236	336.1231	− 1.43	320.0920, 306.0763, 292.0970	CR
29	10.71	Berberine	C_20_H_18_NO_4_^+^	[M]^+^	336.1236	336.1231	− 1.49	321.0982, 320.0920, 306.0763, 292.0970	CR
30	11.11	Worenine	C_20_H_16_NO_4_^+^	[M]^+^	334.1079	334.1074	− 1.50	319.0847	CR
31	11.45	13-methylepiberberine	C_21_H_20_NO_4_^+^	[M]^+^	350.1392	350.1378	− 4.00	334.1074, 320.0920, 306.1126	CR
32	11.45	13-methylberberine	C_21_H_20_NO_4_^+^	[M]^+^	350.1392	350.1378	− 4.00	335.1141, 334.1074, 320.0920, 306.1126	CR
33	16.11	Oxyberberine	C_20_H_17_NO_5_	[M + H]^+^	352.1179	352.1183	1.14	336.0875, 322.0704, 308.0920, 294.0758	CR
34	0.96	Glucose	C_6_H_12_O_6_	[M–H]^−^	179.0561	179.0552	− 5.03	161.0443	SR,CR
35	1.02	Sucrose	C_12_H_22_O_11_	[M–H]^−^	341.1089	341.1089	0.00	179.0559, 161.0448, 113.0239	SR,CR
36	1.07	Quinic acid	C_7_H_12_O_6_	[M–H]^−^	191.0561	191.0559	− 1.05	173.0448, 127.0393	SR,CR
37	1.18	Gallic acid	C_7_H_6_O_5_	[M–H]^−^	169.0142	169.0137	− 2.96	125.0240	SR,CR
38	4.02	Methyl protocatechuate	C_8_H_8_O_4_	[M–H]^−^	167.0350	167.0341	− 5.39	152.0105, 108.0204	SR,CR
39	4.23	protocatechuic acid	C_7_H_6_O_4_	[M–H]^−^	153.0193	153.0182	− 7.19	123.0437, 109.0283, 108.0202	SR,CR
40	4.76	3-hydroxy-4-methoxybenzoic acid 3-O-β-D-glucopyranoside	C_14_H_18_O_9_	[M–H]^−^	329.0878	329.0851	− 8.20	167.0342	CR
41	5.30	Gentisic acid	C_7_H_6_O_4_	[M–H]^−^	153.0193	153.0186	− 4.57	109.0284	SR,CR
42	6.96	3-O-Feruloylquinic acid	C_17_H_20_O_9_	[M–H]^−^	367.1035	367.1017	− 4.90	193.0430, 149.0598, 134.0363	CR
43	6.99	Isoschaftoside	C_26_H_28_O_14_	[M–H]^−^	563.1406	563.1392	− 2.49	473.1083, 383.0759, 353.0650	SR
44	7.72	4-O-Feruloylquinic acid	C_17_H_20_O_9_	[M–H]^−^	367.1035	367.1018	− 4.63	193.0433, 149.0601, 134.0368	CR
45	7.80	Schaftoside	C_26_H_28_O_14_	[M–H]^−^	563.1406	563.1411	0.89	443.0946, 311.0557, 283.0609, 179.0552	SR
46	7.83	Chrysin 6-C-β-D-glucoside 8-C-α-L-arabinoside	C_26_H_28_O_13_	[M–H]^−^	547.1457	547.1454	− 0.55	487.1260, 457.1141, 427.1056, 367.0812, 337.0719	SR
47	8.02	6-Hydroxykaempferol 3-O-β-D-glucoside	C_21_H_19_O_12_	[M–H]^−^	463.0882	463.0887	1.08	303.0510, 257.0452, 153.0182	SR
48	8.03	Carthamidin-7-O-glucoside	C_21_H_20_O_12_	[M–H]^−^	463.0882	463.0886	0.86	287.0558, 269.0450, 181.0132, 166.9925, 161.0232, 153.0184, 129.0026, 113.0230, 119.0490	SR
49	8.09	Acteoside	C_29_H_36_O_15_	[M–H]^−^	623.1981	623.1994	2.09	461.1662, 315.1089, 179.0346, 135.0438	SR
50	8.13	isocarthamidin-7-O-β-D-glucuronide	C_21_H_20_O_12_	[M–H]^−^	463.0882	463.0875	− 1.51	287.0547, 181.0151, 153.0115, 119.0602	SR
51	8.14	Scutellarin	C_21_H_18_O_12_	[M–H]^−^	461.0725	461.0718	− 1.52	285.0396, 164.9952	SR
52	8.21	Viscidulin III-2′-O-β-D-glucoside	C_23_H_24_O_13_	[M–H]^−^	507.1144	507.1138	− 1.18	345.0613, 330.0379, 315.0136	SR
53	8.45	Azelaic acid	C_9_H_16_O_4_	[M–H]^−^	187.0976	187.0968	− 4.28	125.0964	SR,CR
54	8.73	5,2′,6′-Trihydroxy-7,8-dimethoxyflavone-2′-O-β-D-glucoside	C_23_H_24_O_12_	[M–H]^−^	491.1195	491.1190	− 1.02	329.0664, 328.0591, 314.0432, 299.0190	SR
55	8.97	Chrysin 8-C-glucoside	C_21_H_20_O_9_	[M–H]^−^	415.1035	415.1036	0.24	295.0606, 267.0644, 145.0285	SR
56	9.99	Baicalin	C_21_H_18_O_11_	[M–H]^−^	445.0776	445.0774	− 0.45	269.0454, 251.0347, 223.0393, 195.0438, 167.0495, 149.9932	SR
57	10.54	5,7,2′,6′–tetrahydroxyflavanone	C_15_H_12_O_6_	[M–H]^−^	287.0561	287.0558	− 1.05	161.0232, 125.0230	SR
58	10.56	5,7,2′,6′-Tetrahydroxyflavone-2′-O-β-D-glucoside	C_21_H_20_O_11_	[M–H]^−^	447.0933	447.0927	− 1.34	285.0402, 151.0025, 133.0282	SR
59	10.56	Cynaroside	C_21_H_20_O_11_	[M–H]^−^	447.0933	447.0927	− 1.34	285.0402, 267.0295, 213.0551, 163.0025, 151.0025, 133.0282, 125.0230	SR
60	10.57	Oroxylin A-7-O-β-D-glucoside	C_22_H_22_O_10_	[M–H]^−^	445.1140	445.1134	− 1.35	430.0875, 283.0609, 269.0453, 268.0334, 267.0298, 136.9866	SR
61	11.09	Chrysin 7-O-β-D-glucoronide	C_21_H_18_O_10_	[M–H]^−^	429.0827	429.0818	− 2.10	253.0502, 113.0230	SR
62	11.11	Apigenin-7-O-β-D-glucopyranoside	C_21_H_20_O_10_	[M–H]^−^	431.0984	431.0971	− 3.02	269.0440	SR
63	11.17	5,7,2′ -trihydroxy-6-methoxyflavone-7-O-glucuronide	C_22_H_20_O_12_	[M–H]^−^	475.0882	475.0876	− 1.26	299.0559, 284.0324, 283.0244	SR
64	11.29	Dihydroxyflavanone-O-glucoside	C_21_H_20_O_10_	[M–H]^−^	431.0984	431.0981	− 0.70	255.0577, 113.0230	SR
65	11.31	Oroxin A	C_21_H_20_O_10_	[M–H]^−^	431.0984	431.0981	− 0.70	269.0453, 223.0285	SR
66	11.49	Wogonoside	C_22_H_20_O_11_	[M–H]^−^	459.0933	459.0929	− 0.87	283.0608, 268.0370, 175.0237	SR
67	11.54	Tetrahydroxy-methoxyflavone	C_16_H_12_O_7_	[M–H]^−^	315.0510	315.0513	0.95	315.0509, 300.0273, 136.9866	SR
68	11.64	5,7-Dihydroxy-6-methoxyflavone-7-O-glucuronide	C_22_H_22_O_11_	[M–H]^−^	461.1089	461.1090	0.22	285.0765, 270.0531	SR
69	11.66	5, 7-dihydroxy-6, 8-dimethoxyflavone-7-O-glucuronide	C_23_H_22_O_12_	[M–H]^−^	489.1038	489.1031	− 1.43	313.0714, 298.0475, 283.0274	SR
70	11.69	5,7-Dihydroxy-6,8-dimethoxyflavone	C_17_H_14_O_6_	[M–H]^−^	313.0718	313.0714	− 1.28	298.0475, 283.0272, 167.0339	SR
71	11.91	Scutellarein	C_15_H_10_O_6_	[M–H]^−^	285.0405	285.0405	0.00	137.0234, 124.0153, 123.0074	SR
72	12.59	5,2′,5′-trihydroxy-6,7, 8-trimethoxyflavone	C_18_H_16_O_8_	[M–H]^−^	359.0772	359.0774	0.56	344.0528, 329.0315, 229.0139, 133.0284	SR
73	12.86	Apigenin	C_15_H_10_O_5_	[M–H]^−^	269.0455	269.0453	− 0.74	225.0545, 183.0446, 117.0333	SR
74	13.02	Norwogonin	C_15_H_10_O_5_	[M–H]^−^	269.0455	269.0453	− 0.74	269.0453, 241.0497, 223.0397, 197.0600, 195.0443, 169.0649, 139.0025, 136.9866, 111.0078	SR
75	13.13	Viscidulin II	C_17_H_14_O_7_	[M–H]^−^	329.0667	329.0669	0.61	314.0426, 299.0194, 271.0247, 164.9815	SR
76	13.21	Baicalein	C_15_H_10_O_5_	[M–H]^−^	269.0450	269.0453	1.12	251.0340, 241.0504, 223.0394, 195.0443, 167.0492, 149.9946, 121.9982	SR
77	14.38	Trihydroxy-tetramethoxyflavone	C_19_H_18_O_9_	[M–H]^−^	389.0878	389.0879	0.26	374.0645, 149.0234	SR
78	15.33	8,8″-Bibaicalein	C_30_H_18_O_10_	[M–H]^−^	537.0827	537.0823	− 0.74	391.0457, 373.0400, 245.0086	SR
79	15.57	Wogonin	C_16_H_12_O_5_	[M–H]^−^	283.0612	283.0611	− 0.35	268.0374, 163.0016, 109.9997	SR
80	15.66	Chrysin	C_15_H_10_O_4_	[M–H]^−^	253.0506	253.0504	− 0.79	143.0488, 107.0144	SR
81	15.90	Noroxylin	C_19_H_18_O_8_	[M–H]^−^	373.0929	373.0927	− 0.54	358.0693, 343.0448	SR
82	16.08	Oroxylin A	C_16_H_12_O_5_	[M–H]^−^	283.0612	283.0611	− 0.35	268.0375, 224.0474	SR
83	16.70	Tenaxin I	C_18_H_16_O_7_	[M–H]^−^	343.0823	343.0823	0.00	328.0579, 313.0354, 194.9924	SR

**Fig. 3:**
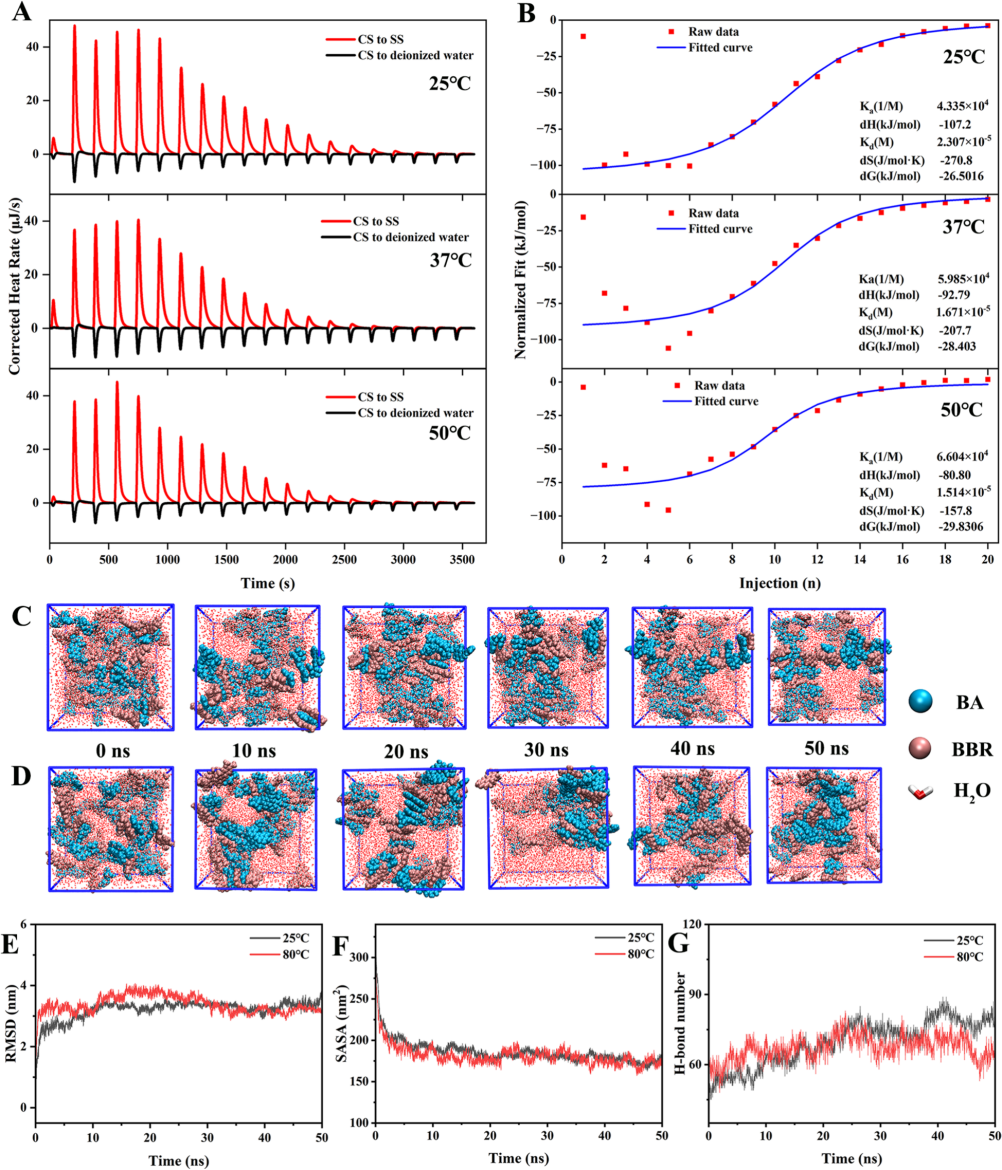
**A** ITC titration curve. **B** ITC fitting curve. **C** Molecular dynamics simulation of self-assembly of BA and BBR in aqueous solution (0–50 ns, 25 ℃). **D** Molecular dynamics simulation of self-assembly of BA and BBR in aqueous solution (0–50 ns, 80 ℃). **E** RMSD varies with time. **F** SASA varies with time. **G** H-bond number

ITC is an efficient tool that can accurately and continuously monitor heat changes during the interaction process, and calculate the key thermodynamic parameters based on these changes. Figure 3A and B showed the energy change curve and thermodynamic parameters, respectively. The blank deduction was performed based on the titration of CS into deionized water, and the curve fitting was performed using Nano Analyze software. At the set temperature, the titration process of CS into deionized water was a dilution process, and the characteristic peak was downward, which was an endothermic reaction. The characteristic peak of CS titrated SS was upward, which was exthermic reaction. The fitted curve exhibited an approximate S-shape, which was the characteristic trend of chemical reactions. Both of them displayed |ΔH| >|–TΔS|. These results indicated that the reaction of SR and CR was a spontaneous chemical reaction driven by enthalpy, rather than a simple physical process. Meanwhile, this suggested that there might be some weak interaction between the acid and basic components of the two medicinal materials. Based on our previous research results, the type of non-covalent bond in this interaction was likely to include hydrogen bond and so on. Titrations with the same stock solution at different temperatures (25, 37 and 50 °C) showed that K_a_ tended to increase with increasing temperature, while K_d_ decreased with increasing temperature, indicating that increasing temperature was beneficial for binding between SR and CR.

Based on the consistency of the supramolecular characteristics between SR-CR and its main components BA and BBR, the supramolecular formation process was further simulated using MD. We analyzed the self-assembly processes of BA and BBR at different temperatures within 50 ns, and found that the supramolecular system formed by self-assembly at 80 ℃ was more closely arranged, which was consistent with the change trend observed by SEM (Fig. 3C, D). Meanwhile, the results of RMSD and SASA indicated that the whole system tended to stabilize after 50 ns (Fig. 3E, F). In order to analyze the driving force in the self-assembly process, the number of intermolecular hydrogen bonds in the simulation process was statistically analyzed, and it was found that the number of hydrogen bonds formed by the supramolecular system at 80 ℃ was more than that at 25 ℃, which was 80.07 and 73.61 respectively (Fig. 3G). Then, the representative interaction energy of the self-assembled system was analyzed. Clou-SR represented the short-range Coulomb interaction, including hydrogen bond, etc. LJ-SR stands for van der Waals energy and mainly includes various conjugations. The results showed that E_(Coul-SR)_ was lower than E_(LJ-SR)_ at different temperatures, indicating that the interaction between the self-assembled systems was dominated by hydrogen bonding. The E_(Coul-SR)_ at 80℃ was lower, indicating that the hydrogen bond between molecules was stronger (Table 2). Based on the above experimental results, stronger bonding ability, more hydrogen bonds and stronger forces under heating conditions led to the assembly more closely arranged, which might be one of the reasons for the structural changes in supramolecular systems at the microscopic level.

**Table 2:** The short-range coulombic interaction energy E_(Coul-SR)_ and the short-range Lennard–Jones energy E_(LJ-SR)_ (kJ mol^−1^) of self-assembly process

Temperature (℃)	E_(Coul-SR)_ (kJ·mol^−1^)	E_(LJ-SR)_ (kJ·mol^−1^)
25	− 1.43 × 10^4^	− 7.32 × 10^3^
80	− 1.46 × 10^4^	− 7.12 × 10^3^

Based on the above experimental results, we conjectured that the interaction between SR and CR was regulated by thermal energy, and heating was conducive to the binding of SR and CR. The influence of temperature would further show many differences in the morphology of the supramolecular system, such as the transformation of the microscopic morphology of the formed supramolecular system from NFs to NPs.

### Characterization of antibacterial activity in vitro

Bacteria that can be used in probiotic preparations (*B. subtilis* and *E. faecium*) and pathogenic bacteria (*S. aureus*) were selected for antibacterial experiments in vitro to further explore the relationship between the structural differences of supramolecules and biological activity. Considering that SS exhibited negligible antibacterial activity at the tested concentrations, CS was selected as the positive control to evaluate the changes in the antibacterial activity of the samples. Meanwhile, the solvent had no significant effect on the growth of the bacteria (Fig S3).

**Fig. 4:**
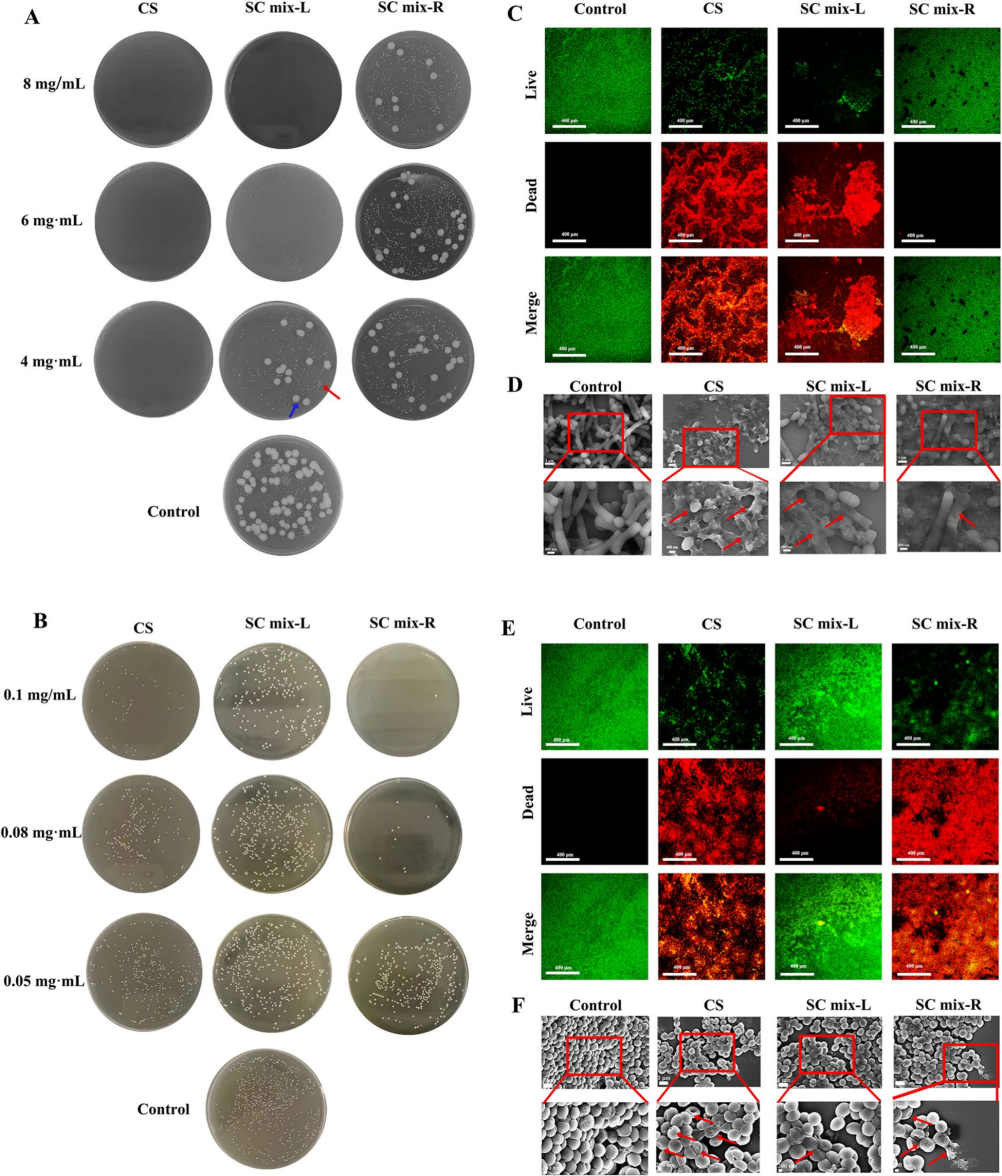
Characterization of antibacterial activity *in vitro*. **A** Plate coating of *E. faecium* and *B. subtilis*. **B** Plate coating of *S. aureus*. Live and dead bacteria staining of *B. subtilis and E. faecium* (**C**) and *S. aureus* (**E**) (Red fluorescence indicates dead bacteria, green fluorescence indicates living bacteria). SEM images of *B. subtilis and E. faecium* (**D**) and *S. aureus* (**F**)

As shown in Fig. 4A, CS had obvious inhibitory effect on *B. subtilis* and *E. faecium* under the set concentration conditions, and no obvious colony growth was observed. After the mechanical mixing of SR and CR, the inhibitory effect was reduced. At 4 mg·mL^−1^, both SC mix-L and SC mix-R groups had obvious colonies of *E. faecium* (indicated by the red arrow) and *B. subtilis* (indicated by the blue arrow) on the plates. At 6 mg·mL^−1^, SC mix-L group had only *E. faecium* colonies compared with SC mix-R. The opposite results were obtained in the in vitro antibacterial experiments against *S. aureus* (Fig. 4B). CS had obvious inhibitory effect at 0.1 mg·mL^−1^, and only a few colonies could be observed. After the mechanical mixing of SR and CR, the inhibitory effect was different. At 0.1·mg mL^−1^, obvious colonies were observed in SC mix-L group, indicating that the inhibitory activity was significantly weakened. On the contrary, the antibacterial activity increased after heating, and there were less colonies growth in SC mix-R group compared with CS group.

Live and dead bacteria staining was used to further evaluate the differences in antibacterial activity. At the same time, SEM was used to observe the morphological changes of different bacteria treated with the same concentration of samples. Green fluorescence represents live bacteria, and red fluorescence represents dead bacteria. For *B. subtilis and E. faecium* (Fig. 4C), in SC mix-L group and CS group, obvious red fluorescence could be seen. Obvious green fluorescence could be seen in the Control group and SC mix-R group. As shown in Fig. 4D, the bacteria in the control group had smooth surface and complete structure. In the CS group, the integrity of the bacteria was seriously damaged, with significant morphological changes and even extensive rupture. The CS mix-L group also showed obvious bacterial shrinkage, indicating that the integrity of the bacteria was damaged. At the same time, the bacteria were observed to be tightly wound by NFs. In the CS mix-R group, only a small number of bacteria were found crumpled, indicating that the integrity of the bacteria was damaged. For *S. aureus* (Fig. 4E), in SC mix-R group and CS group, obvious red fluorescence could be seen. And obvious green fluorescence could be seen in the Control group and SC mix-L group. As shown in Fig. 4F, in the control group the bacteria also had smooth surface and complete structure. In the CS mix-L group, the surface of the bacteria was wrinkled, but the damage was less severe. But the integrity of the bacteria was seriously damaged with significant morphological changes and even extensive rupture in the CS mix-R group, which was more serious than the CS group.

**Fig. 5:**
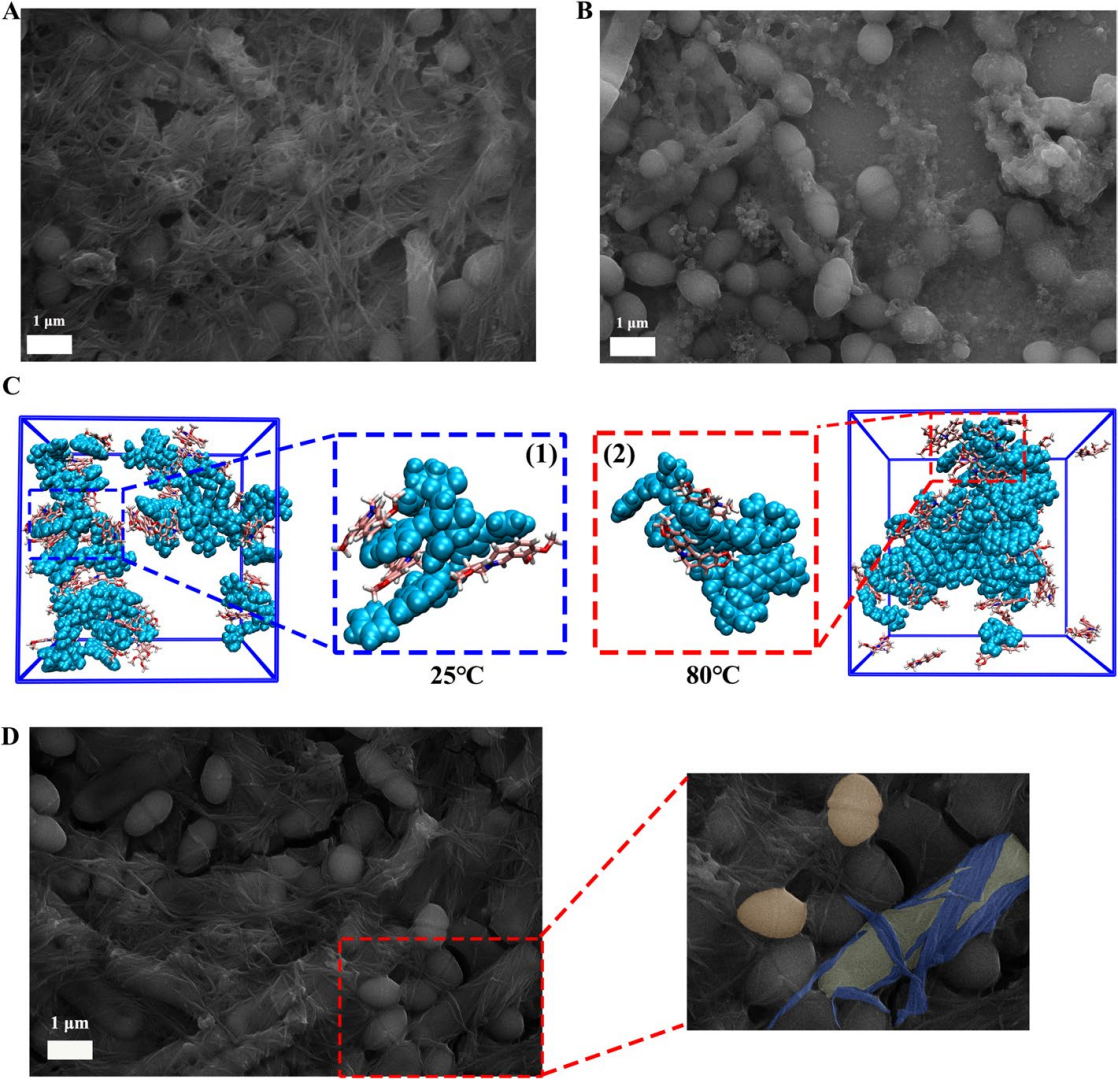
Interactions between bacteria and supramolecules. **A** *B. subtilis and E. faecium* with NFs. **B** *B. subtilis and E. faecium* with NPs. **C** The arrangement of molecules in NFs (1) and NPs (2). **D** *B. subtilis* was tightly wrapped by NFs

At the same time, we observed that the structural integrity of supramolecules was maintained when interacting with bacteria (Fig. 5A, B). Structural integrity implied that the internal arrangement of the supramolecules remained intact. However, the antibacterial activity of same supermolecule against different bacteria showed selectivity. The structure of the supramolecular system and the shape of the bacteria might account for the different antibacterial activities. BBR was considered to be the main pharmacodynamic component in the drug pair that produces antimicrobial activity. Further combining the results of MD simulations (Fig. 5C), we found that in NPs, there were more BBR exposed. But in NFs, BA and BBR molecules were interleaved. The zeta potential value of BA-BBR mix-R was positive and larger than that of BA-BBR mix-L, which further supported this conclusion [38]. This might be one of the reasons for the better antibacterial activity of NPs against *S. aureus.* For the opposite antibacterial effects of the same supramolecular system against different bacteria, it might be closely related to the shape of the bacteria. From the results of plate coating and SEM (Fig. 5D), we could find that for *B. subtilis* and *E. faecium*, the antibacterial effect of NFs on *B. subtilis* is better. The shrinkage of *B. subtilis* was more obvious, while most of *E. faecium* remained intact. It was also observed that *B. subtilis* as rod-shape bacteria could be more tightly encapsulated by NFs, especially compared to *E. faecium as* spherical bacteria. *B. subtilis* and *E. faecium* could rapidly consume oxygen to provide a hypoxic environment for the growth of anaerobic intestinal probiotics such as *Bifidobacterium* and competitively inhibit the growth of pathogens [39], which might be the reason why the herb pair SR and CR were often decocted together in clinical use. The supramolecular system formed after heating could enhance the inhibitory effect on pathogenic bacteria such as *S. aureus* and alleviate the damage to probiotics at the same time. Meanwhile, the results of our previous experiments also confirmed that NPs were more effective than NFs in removing the biofilm of *S. aureus* [25]. However, the in-depth biological mechanism differences need to be further studied. To sum up, the above research showed that thermal energy could change the activity of the CHMs by influencing the structure of supermolecules.

**Fig. 6:**
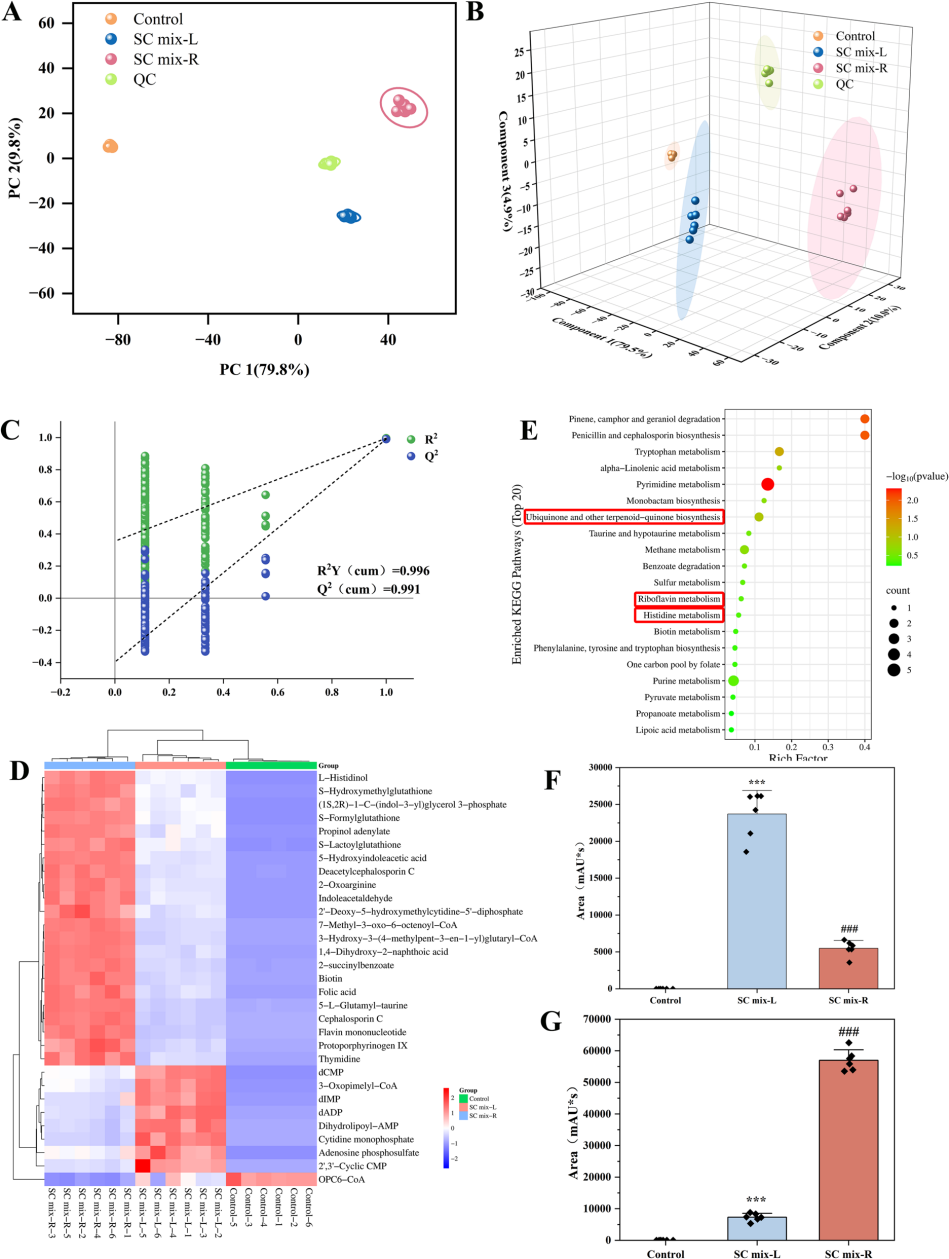
Non-targeted metabonomics analysis for *E. faecium and B. subtilis*. **A** PCA of metabolites. **B** PLS-DA score plot. **C** Permutation plots of the PLS-DA models. **D** Hierarchical clustering results of 31 metabolites. **E** Pathway analysis of 31 potential biomarkers. Abundance levels of metabolites dADP (**F**) and Flavin mononucleotide (**G**). ^***^p < 0.001, ^**^p < 0.01, ^*^p < 0.05 compared with Control group; ^###^*p* < 0.001, ^##^*p* < 0.01, ^#^*p* < 0.05 compared with SC mix-L group

Furthermore, untargeted metabolomics was used to explore the effect of supramolecular transition on the antibacterial mechanism of the samples. For *B. subtilis and E. faecium,* the reliability of the metabolomics data was first validated by assessing the tight clustering of all QC samples in the PCA score plot (Fig. 6A), indicating excellent instrumental stability and experimental reproducibility. PLS-DA results showed that SC mix-R group and SC mix-L group were significantly separated from the Control group (Fig. 6B). Permutation tests (n = 200) (Fig. 6C) confirmed the validity of the models without overfitting. Comparative screening of SC mix-L vs Control and SC mix-R vs SC mix-L identified 31 differential metabolites (Table 3). Notably, 8 metabolites exhibited inverse regulation trends between SC mix-L vs Control and SC mix-R vs SC mix-L comparisons. Hierarchical clustering heatmaps displayed clear segregation among the Control group, SC mix-L group and SC mix-R group (Fig. 6D). The screened differential metabolites were mapped to KEGG pathways and enrichment analysis was performed. Pathways with impact values over 0.1 were considered as candidate target pathways. Among them, three metabolic pathways (Fig. 6E), namely ubiquinone and other terpenoid-quinone biosynthesis (impact = 0.2745), riboflavin metabolism (impact = 0.13981) and histidine metabolism (impact = 0.10385) were considered to be the main pathways. Histidine is one of the amino acids that can be converted into an intermediate of the tricarboxylic acid (TCA) cycle. Ubiquinone [40] and riboflavin [41] were essential for bacterial energy metabolism. Specifically, after the treatment with NFs, the conversion of dADP was affected, and the abundance level significantly increased, thereby influencing the energy metabolism of the bacteria (Fig. 6F). The flavin mononucleotide participates in the TCA cycle and the electron transfer in the respiratory chain. Compared to the treatment with NPs, the expression level of flavin mononucleotide significantly decreased after NFs treatment, reflecting the energy crisis state of the bacteria (Fig. 6G). These results indicated that NFs could inhibit bacterial growth and reproduction by interfering with bacterial energy metabolism.

**Table 3:** Databases of 31 potential differential metabolites for *E. faecium* and B*. subtilis*

Compound name	KEGG ID	HMDB	PubChem	Control *vs* SC mix-L			SC mix-R *vs* SC mix-L		
				FC	p value	Trend	FC	p.value	Trend
L-Histidinol	C00860	HMDB0003431	165271	4.967	6.5288 × 10^–12^	Up	2.0843	3.8688 × 10^–9^	Up
2-Oxoarginine	C03771	HMDB0004225	558	6.1563	3.0269 × 10^–10^	Up	2.7362	5.1853 × 10^–10^	Up
1,4-Dihydroxy-2-naphthoic acid	C03657	HMDB0244212	671	13.759	1.9019 × 10^–08^	Up	3.4139	2.667 × 10^–11^	Up
Indoleacetaldehyde	C00637	HMDB0001190	800	8.4486	1.2638 × 10^–08^	Up	2.6795	7.7811 × 10^–9^	Up
5-Hydroxyindoleacetic acid	C05635	HMDB0000763	1826	35.828	2.8058 × 10^–10^	Up	2.6609	6.8887 × 10^–11^	Up
Thymidine	C00214	HMDB0000273	5789	4.4926	4.8131 × 10^–6^	Up	3.5969	1.053 × 10^–7^	Up
2-succinylbenzoate	C02730	HMDB0304087	955	2.0046	2.161 × 10^–6^	Up	3.4951	1.4513 × 10^–10^	Up
Biotin	C00120	HMDB0000030	171548	3.354	1.9913 × 10^–8^	Up	3.8301	5.7287 × 10^–10^	Up
5-L-Glutamyl-taurine	C05844	HMDB0004195	68759	169.77	1.4713 × 10^–9^	Up	6.0751	1.3918 × 10^–12^	Up
Protoporphyrinogen IX	C01079	HMDB0001097	121893	2.3263	1.6641 × 10^–5^	Up	4.5478	1.1565 × 10^–6^	Up
(1S,2R)−1-C-(indol-3-yl) glycerol 3-phosphate	C03506	HMDB0303952	444150	111.21	1.4787 × 10^–11^	Up	2.1517	1.6111 × 10^–10^	Up
2',3'-Cyclic CMP	C02354	METPA0278	NA	785.01	2.8866 × 10^–5^	Up	0.23722	2.6977 × 10^–4^	Down
dIMP	C06196	HMDB0006555	91531	2.0375	7.1473 × 10^–7^	Up	0.31428	2.7409 × 10^–6^	Down
dCMP	C00239	HMDB0001202	13945	23816	5.9493 × 10^–11^	Up	0.39028	1.8147 × 10^–8^	Down
Cytidine monophosphate	C00055	HMDB0000095	6131	2.0611	1.4931 × 10^–6^	Up	0.14313	1.6022 × 10^–8^	Down
S-Formylglutathione	C01031	HMDB0001550	189122	398.95	3.4381 × 10^–9^	Up	2.0489	1.1348 × 10^–8^	Up
dADP	C00206	HMDB0001508	188966	2.1516	1.1024 × 10^–6^	Up	0.20934	3.9546 × 10^–8^	Down
S-Hydroxymethylglutathione	C14180	HMDB0004662	447123	2.1968	3.05 × 10^–6^	Up	2.2657	2.4802 × 10^–8^	Up
Deacetylcephalosporin C	C03112	METPA0361	NA	6.0534	2.7182 × 10^–10^	Up	2.8717	2.6458 × 10^–10^	Up
Adenosine phosphosulfate	C00224	HMDB0001003	10238	2.0104	6.4293 × 10^–5^	Up	0.45729	7.5499 × 10^–5^	Down
S-Lactoylglutathione	C03451	HMDB0001066	440018	42.682	3.3954 × 10^–8^	Up	2.1786	4.6472 × 10–7	Up
Propinol adenylate	C05983	HMDB0006806	440863	2.3963	4.4638 × 10^–5^	Up	2.2392	1.218 × 10^–7^	Up
Cephalosporin C	C00916	HMDB0060450	65536	2.548	1.1491 × 10^–5^	Up	6.727	2.8216 × 10^–10^	Up
Folic acid	C00504	HMDB0000121	6037	2.1976	5.4544 × 10^–6^	Up	3.5855	1.7042 × 10^–08^	Up
2′-Deoxy-5-hydroxymethylcytidine-5′-diphosphate	C11038	METPA0984	NA	2.1514	3.5565 × 10^–6^	Up	2.8291	1.004 × 10^–6^	Up
7-Methyl-3-oxo-6-octenoyl-CoA	C16466	HMDB0060421	9549325	64.3	1.4011 × 10^–10^	Up	3.6861	9.5799 × 10^–12^	Up
3-Oxopimelyl-CoA	C06715	HMDB0012158	441147	855.35	8.203 × 10^–11^	Up	0.33525	3.0623 × 10^–8^	Down
Flavin mononucleotide	C00061	HMDB0001520	643976	2.3165	5.3716 × 10^–6^	Up	7.0857	4.8933 × 10^–11^	Up
3-Hydroxy-3-(4-methylpent-3-en-1-yl) glutaryl-CoA	C04675	HMDB0060372	11966138	1286	2.6345 × 10^–9^	Up	4.0078	5.0001 × 10^–11^	Up
Dihydrolipoyl-AMP	C22161	NA	NA	2.1673	1.0685 × 10^–6^	Up	0.17766	3.7979 × 10^–8^	Down
OPC6-CoA	C16331	HMDB0011114	53480660	0.18198	4.1762 × 10^–8^	Down	0.2298	1.5666 × 10^–3^	Down

**Fig. 7:**
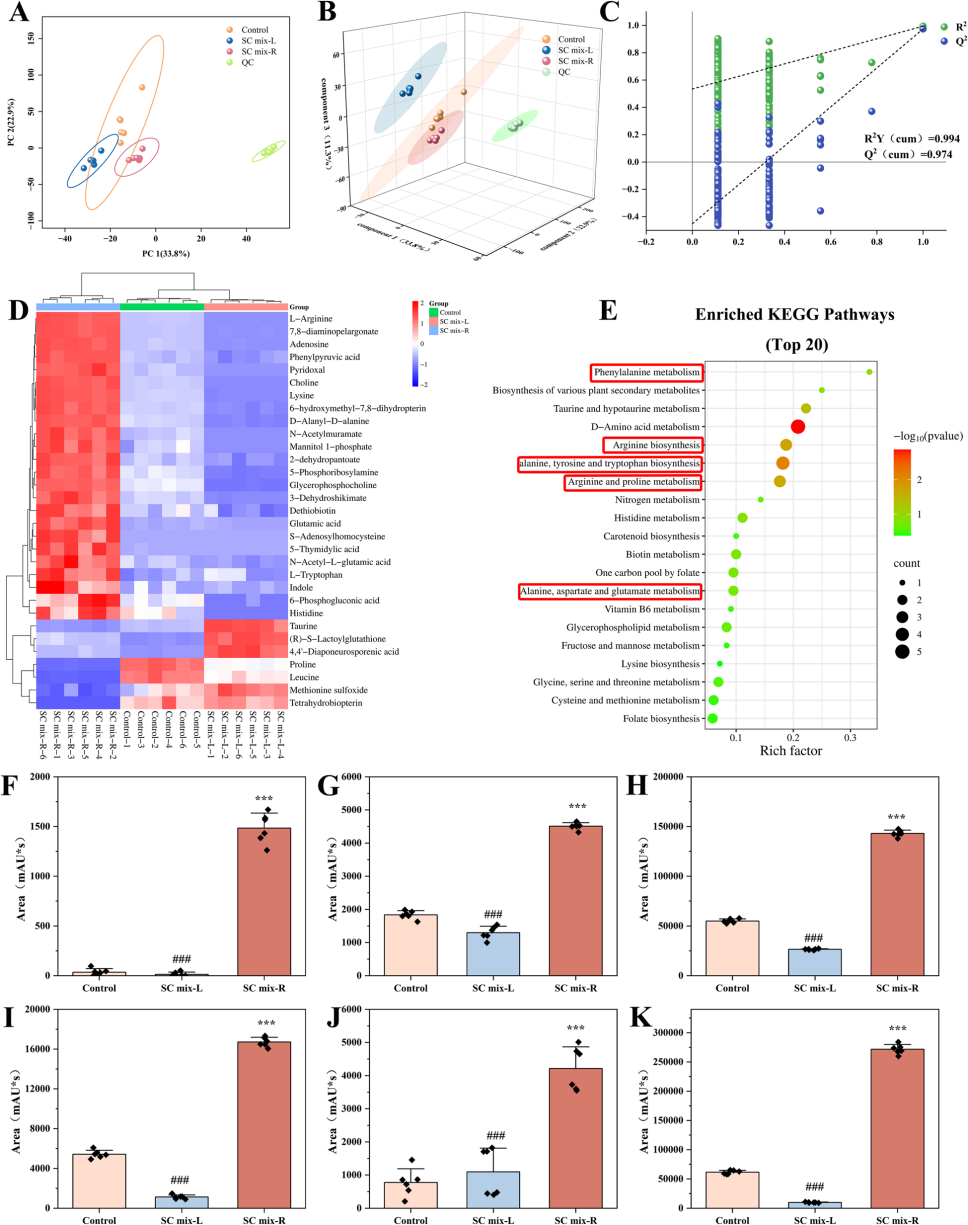
Non-targeted metabonomics analysis for *S. aureus*. **A** PCA of metabolites. **B** PLS-DA score plot. **C** Permutation plots of the PLS-DA models. **D** Hierarchical clustering results of 31 metabolites. **E** Pathway analysis of 31 potential biomarkers. Abundance levels of metabolites Glutamic acid (**F**), Lysine (**G**), l-Tryptophan (**H**), Phenylpyruvic acid (**I**), d-Alanyl-d-alanine (**J**) and l-Arginine (**K**). ^***^p < 0.001, ^**^p < 0.01, ^*^p < 0.05 compared with Control group; ^###^*p* < 0.001, ^##^*p* < 0.01, ^#^*p* < 0.05 compared with SC mix-R group

The metabolomic analysis data of *S. aureus* demonstrated high reproducibility, as evidenced by the tight clustering of QC samples in the PCA score plot (Fig. 7A). In the PLS-DA analysis (Fig. 7B), the SC mix-R group was significantly separated from the Control group, while the distribution area of the SC mix-L group was close to that of the Control group, which was highly consistent with the previous antibacterial activity data. Permutation tests (Fig. 7C) (n = 200) confirmed the statistical model had a good fit and predictive ability without overfitting. Comparative analysis of SC mix-R vs Control and SC mix-L vs SC mix-R identified 31 differential metabolites (Table 4). In the SC mix-R vs Control comparison, the levels of 24 metabolites were significantly upregulated and 5 downregulated, while the SC mix-L vs SC mix-R comparison exhibited an inverse regulatory trend. The opposite regulatory trend further indicated that the differences of supramolecular system had different effects on the metabolism of *S. aureus*. Heatmaps of 31 metabolites were constructed and visualized. As shown in Fig. 7D, the metabolites in the Control group, SC mix-L group and SC mix-R group achieved distinct clusters. KEGG enrichment analysis showed that five pathways, namely phenylalanine metabolism (impact = 1), arginine biosynthesis (impact = 0.32427), alanine, aspartate and glutamate metabolism (impact = 0.32075), arginine and proline metabolism (impact = 0.21429), alanine, tyrosine and tryptophan biosynthesis (impact = 0.10547) were considered to be the main pathways (Fig. 7E). Further statistical analysis of the abundance levels of various metabolites revealed that the abundance levels of amino acids significantly increased after NPs treatment, showing different abundance changes compared to those after NFs treatment (Fig. 7F–K). These results suggested that NPs could inhibit the growth of bacteria by affecting the biosynthesis and metabolism of amino acids. Collectively, our results demonstrated that thermodynamically driven morphology transition mediated selective antibacterial effects against different bacteria through modulation of core metabolic pathways.

**Table 4:** Databases of 31 potential differential metabolites for *S. aureus*

Compound name	KEGG ID	HMDB	PubChem	Control *vs* SC mix-R			SC mix-L *vs* SC mix-R		
				FC	p. value	Trend	FC	p. value	Trend
Histidine	C00025	HMDB0000148	33032	39.662	5.73 × 10^–10^	Up	0.01196	4.30 × 10^–10^	Down
Choline	C00114	HMDB0000097	305	4.108	1.28 × 10^–13^	Up	0.025198	9.49 × 10^–15^	Down
2-dehydropantoate	C00966	HMDB0304068	NA	3.3728	2.77 × 10^–9^	Up	0.1277	4.58 × 10^–10^	Down
Indole	C00463	HMDB0000738	798	6.5204	3.96 × 10^–4^	Up	0.068434	1.14 × 10^–4^	Down
Phenylpyruvic acid	C00166	HMDB0000205	997	2.455	2.15 × 10^–12^	Up	0.28765	7.33 × 10^–12^	Down
Taurine	C00245	HMDB0000251	1123	0.44106	6.17 × 10^–8^	Down	9.1639	1.30 × 10^–10^	Up
3-dehydroshikimate	C02637	HMDB0304122	NA	3.7087	1.64 × 10^–8^	Up	0.015077	4.52 × 10^–10^	Down
Lysine	C00047	HMDB0000182	5962	2.6034	7.54 × 10^–14^	Up	0.18517	9.67 × 10^–16^	Down
Proline	C00148	HMDB0000162	145742	0.17112	2.08 × 10^–13^	Down	3.3359	3.80 × 10^–13^	Up
D-Alanyl-D-alanine	C00993	HMDB0003459	5460362	3.0781	7.24 × 10^–13^	Up	0.06782	4.83 × 10^–15^	Down
Pyridoxal	C00250	HMDB0001545	1050	5.8814	2.74 × 10^–10^	Up	0.19102	7.25 × 10^–11^	Down
2-Amino-4-hydroxy-6-hydroxymethyl-7,8-dihydropteridine	C01300	METPA0169	NA	3.4986	1.26 × 10^–11^	Up	0.053946	1.23 × 10^–12^	Down
Leucine	C00123	HMDB0000687	6106	0.10599	1.92 × 10^–12^	Down	6.1197	1.13 × 10^–9^	Up
Histidine	C00135	HMDB0000177	6274	2.0452	4.525 × 10^–3^	Up	0.11778	2.55 × 10^–5^	Down
L-Tryptophan	C00078	HMDB0000929	6305	5.435	7.48 × 10^–7^	Up	0.25995	1.38 × 10^–5^	Down
N-Acetyl-L-glutamic acid	C00624	HMDB0001138	70914	3.3294	1.51 × 10^–06^	Up	0.17485	1.20 × 10^–7^	Down
L-Arginine	C00062	HMDB0000517	6322	4.4213	4.89 × 10^–14^	Up	0.036141	2.75 × 10^–15^	Down
5-Phosphoribosylamine	C03090	HMDB0001128	439905	2.8192	3.96 × 10^–9^	Up	0.018941	2.20 × 10^–11^	Down
Methionine sulfoxide	C02989	HMDB0002005	158980	0.33562	1.14 × 10^–4^	Down	4.5651	1.28 × 10^–8^	Up
7,8-Diaminononanoate	C01037	METPA0113	NA	3.3823	6.07 × 10^–15^	Up	0.14128	8.24 × 10^–16^	Down
Tetrahydrobiopterin	C00272	HMDB0000027	44257	0.17079	2.83 × 10^–06^	Down	5.962	2.72 × 10^–9^	Up
Dethiobiotin	C01909	HMDB0003581	445027	2.2769	5.39 × 10^–6^	Up	0.26678	1.33 × 10^–6^	Down
Mannitol 1-phosphate	C00644	HMDB0001530	130418	4.2995	3.11 × 10^–07^	Up	0.16895	2.78 × 10^–9^	Down
Glycerophosphocholine	C00670	HMDB0000086	657272	2.5611	3.19 × 10^–12^	Up	0.001806	1.51 × 10^–16^	Down
6-Phosphogluconic acid	C00345	HMDB0001316	91493	2.3413	2.917 × 10^–3^	Up	0.045207	5.67 × 10^–5^	Down
Adenosine	C00212	HMDB0000050	60961	4.423	5.59 × 10^–14^	Up	0.048117	2.26 × 10^–15^	Down
N-Acetylmuramate	C02713	HMDB0060493	5462244	5.0333	2.57 × 10^–9^	Up	0.17998	3.25 × 10^–10^	Down
5-Thymidylic acid	C00364	HMDB0001227	9700	129.4	3.60 × 10^–8^	Up	0.14919	1.46 × 10^–7^	Down
S-Adenosylhomocysteine	C00021	HMDB0000939	439155	7.1392	4.05 × 10^–7^	Up	0.14007	4.05 × 10^–7^	Down
S-Lactoylglutathione	C03451	HMDB0001066	440018	4.4819	3.37 × 10^–7^	Up	3.8545	6.83 × 10^–11^	Up
4,4′-Diaponeurosporenic acid	C16146	METPA1831	NA	5.5712	1.16 × 10^–7^	Up	5.9177	4.39 × 10^–10^	Up

## Conclusion

In this study, we found that the binding ability between SR and CR was temperature-dependent. The mechanical mixing of SR and CR, BA and BBR could self-assemble to form NFs. Under the driving of thermal energy, the supramolecular arrangement was changed to form denser NPs. The results of antibacterial experiments showed that with the morphology transition, the damage to bacteria was affected, manifested as selective damage to *S. aureus*, *B. subtilis* and *E. faecium*, and the formed supramolecules maintained their shape during the interaction with bacteria. This study focused on the morphology transition of SR-CR under the thermal energy, and revealed the scientific connotation of the CHMs compatibility to a certain extent. The existence form of supramolecular system was related to the biological activity of CHMs. This work provided evidence for its scientific and effective application in clinical practice, and provided methodological guidance for the development of nanomedicine derived from CHMs. In the future research, we would utilize techniques like gut microbiota sequencing to systematically assess the impact of these supramolecular transitions on overall microbial community balance and homeostasis, which is crucial for understanding their broader physiological effects. Additionally, while the self-assembly of key components (BA, BBR) was demonstrated, the potential roles and interactions of other complex constituents commonly present in CHMs, such as polysaccharides and proteins, in modulating the self-assembly process and the final nanostructures’ properties remain unexplored and warrant detailed investigation.

## Supplementary Information

Supplementary Material 1

## Data Availability

Data is provided within the manuscript or supplementary information files.

## Abbreviations

BA: Baicalin
BBR: Berberine
CHMs: Chinese Herbal Medicines
CR: *Coptidis Rhizoma*
FC: Fold change
NFs: Nanofibers
NPs: Nanospheres
PCA: Principal component analysis
PLS-DA: Partial least squares-discriminant analysis
SR: *Scutellariae Radix*
VIP: Importance in projection
