# Supplementary material for: Thermodynamic-driven supramolecular transition from nanofibers to nanospheres: morphology-dependent antibacterial specificity of herb medicines
Source: Chin Med. 2025 Sep 25;20:147. doi: 10.1186/s13020-025-01185-z (PMC12462035; doi:10.1186/s13020-025-01185-z)
Supplement: Supplementary file 1 — Supplementary Material 1 [file 13020_2025_1185_MOESM1_ESM.docx]

**Supplementary material**

**Materials and methods**

*HPLC characterization of BA in SC mix-L and SC mix-R*

A high-performance liquid chromatography (Agilent Technologies, USA) method was used to compare the BA of SC mix-L and SC mix-R. Equipping with an Ultimate LP-C18 column (4.6 mm × 250 mm, 5 μm), being maintained at 25°C. The flow rate of mobile phase was maintained at 1.0 mL/min with 0.2% (v/v) aqueous phosphoric acid solution (A) and acetonitrile (B). The gradient elution conditions were set as follows: 0-10 min, 5%-15% B; 10-18 min, 15%-23% B; 18-38 min, 23%-35% B; 38-45 min, 35%-40% B; 45-50 min, 40%-90% B; 50-60 min, 90% B; 60-62 min, 90%-5% B; 62-68 min, 5% B. The injection volume was 10 μL. The detection wavelength is 280 nm.

*HPLC characterization of BBR in SC mix-L and SC mix-R*

A high-performance liquid chromatography (Agilent Technologies, USA) method was used to compare the BBR of SC mix-L and SC mix-R. Equipping with an Ultimate LP-C18 column (4.6 mm × 250 mm, 5 μm), being maintained at 25°C. The flow rate of mobile phase was maintained at 1.0 mL/min with 0.1% (v/v) aqueous phosphoric acid solution (A) and methanol (B). The gradient elution conditions were set as follows: 0-30 min, 30% B; 30-31 min, 30%-98% B; 31-51 min, 98% B; 51-65 min, 98%-30% B; 45-50 min, 40%-90% B; 50-60 min, 90% B The injection volume was 2 μL. The detection wavelength is 254 nm and 354 nm..

*Non-targeted metabonomics analysis*

After drying, 200 μL was added to each liquid phase vial, and 6 bottles were made in parallel, and measured on a liquid mass spectrometry instrument (UHPLC-Q-Orbitrap HRMS). Column: Acquity UPLC HSS T3 (2.1 mm × 100 mm, 1.8 μm, Agilent) was used. The mobile phase consisted of 0.1 % (v/v) aqueous formic acid solution (A) and methanol (B). The gradient elution conditions were 0-2 min, 80-70% B; 2-5 min, 70-55% B; 5-6.5 min, 55-40% B; 6.5-12 min, 40-35% B; 12-14 min, 35-15% B; 14-18 min, 15-0% B; 18-18.1 min, 0-80% B; 18.1-20 min, 80% B. The flow rate was 0.3 mL/min. The ion source adopted ESI to collect information in positive (ESI^+^) electrospray ionization mode (EM ACE600, Leica Technology Co., LTD.). The dry gas was the nitrogen gas. The capillary and auxiliary gas heater temperatures were both set to 350°C.

**Results and discussion**


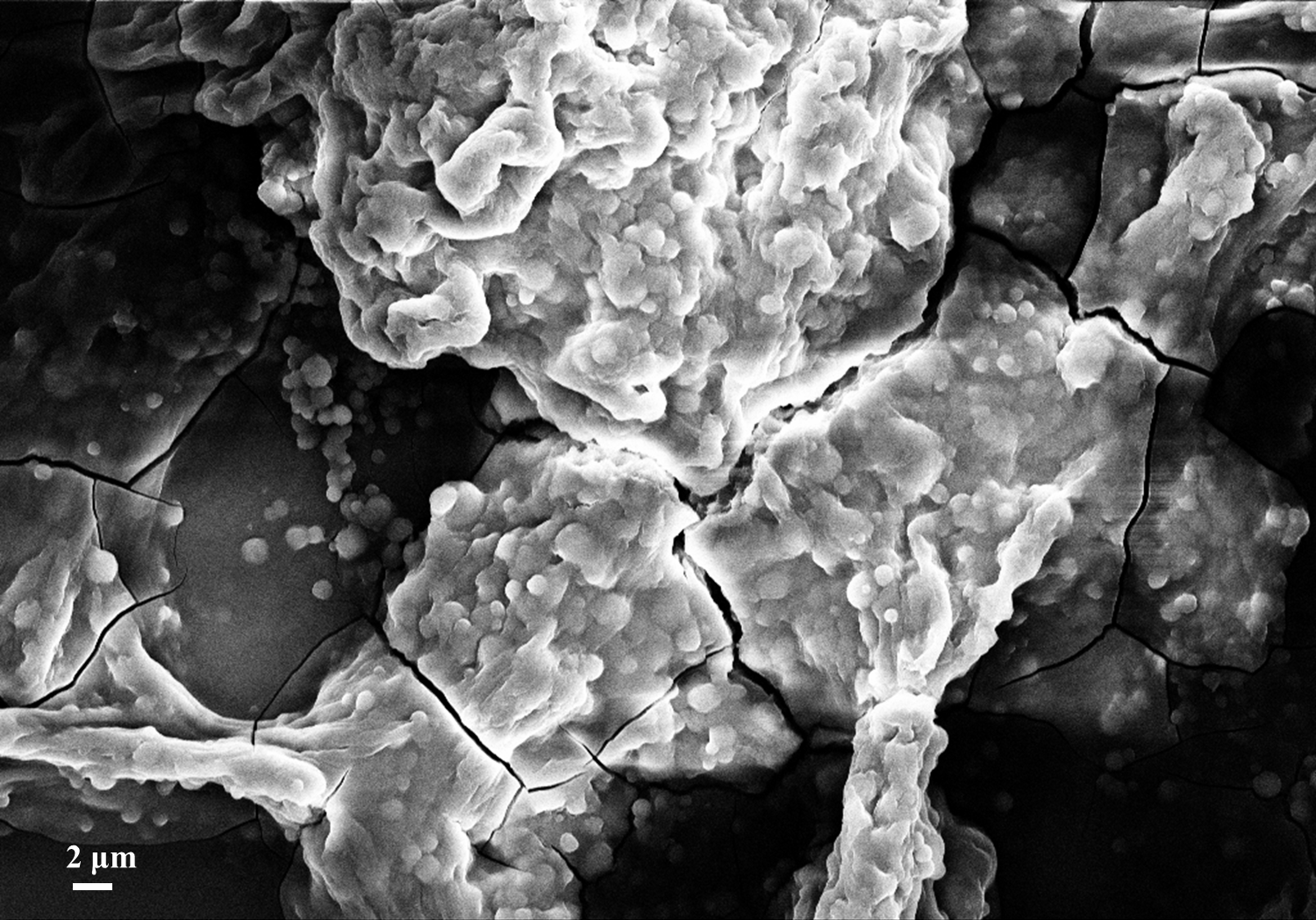


Fig. S1 A transition state between NFs and NPs





Fig. S2 HPLC characterization of BA and BBR in SC mix-L and SC mix-R.

A: comparison of BA in SC mix-L and SC mix-R; B: peak area of BA;

C: comparison of BBR in SC mix-L and SC mix-R; D: peak area of BBR.


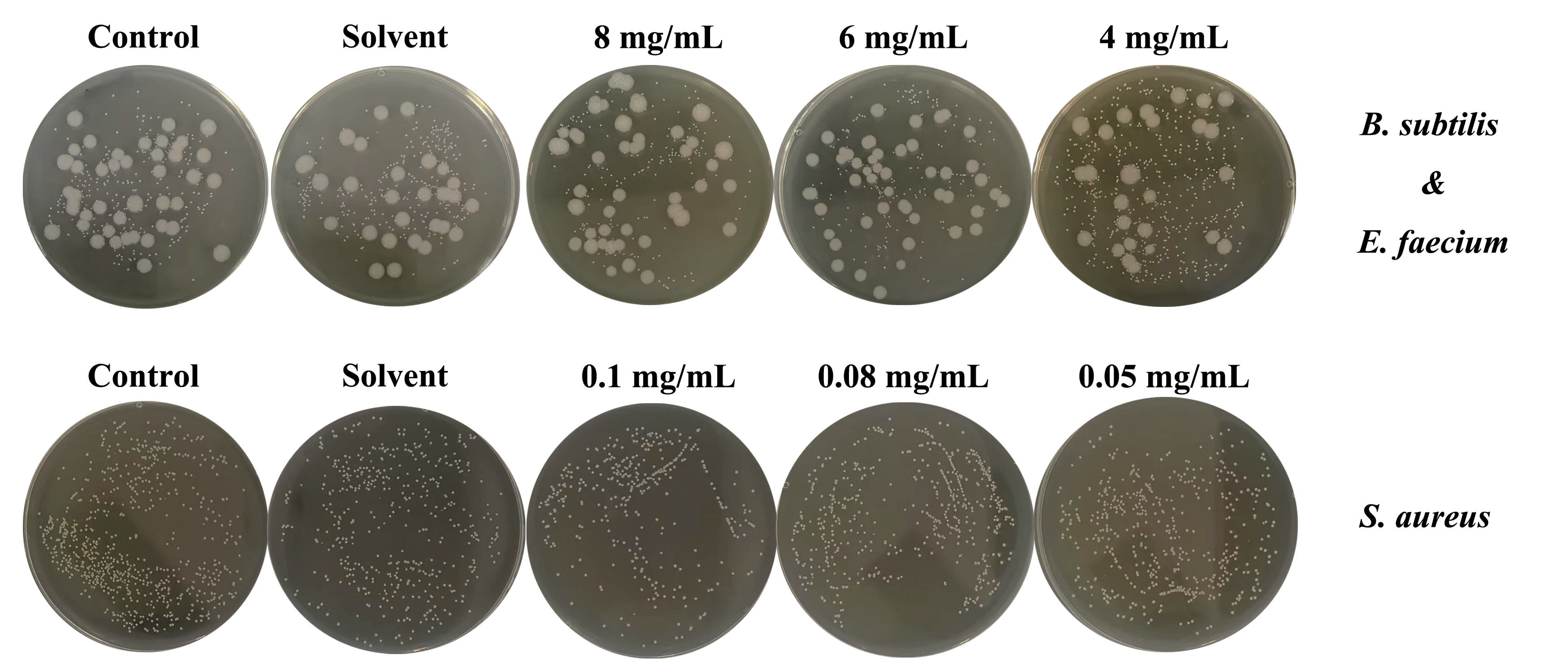


Fig. S3 The solvent has no significant effect on the growth of bacteria and SS exhibited negligible antibacterial activity at the tested concentrations.
